# Phylogenomic and phenotypic analyses highlight the diversity of antibiotic resistance and virulence in both human and non-human *Acinetobacter baumannii*

**DOI:** 10.1128/msphere.00741-23

**Published:** 2024-03-05

**Authors:** Ellen M. E. Sykes, Valeria Mateo-Estrada, Raelene Engelberg, Anna Muzaleva, George Zhanel, Jeremy Dettman, Julie Chapados, Suzanne Gerdis, Ömer Akineden, Izhar U. H. Khan, Santiago Castillo-Ramírez, Ayush Kumar

**Affiliations:** 1 Department of Microbiology, University of Manitoba, Winnipeg, Manitoba, Canada; 2 Programa de Genómica Evolutiva, Centro de Ciencias Génomicas, Universidad Nacional Autónoma de México, Cuernavaca, Mexico; 3 Department of Medical Microbiology and Infectious Diseases, University of Manitoba, Winnipeg, Manitoba, Canada; 4 Ottawa Research and Development Centre (ORDC), Agriculture and Agri-Food Canada, Ottawa, Ontario, Canada; 5 Dairy Sciences, Institute of Veterinary Food Science, Justus-Liebig, University of Giessen, Giessen, Germany; University of Napoli Federico II, Napoli, Italy

**Keywords:** *Acinetobacter baumannii*, antibiotic resistance genes, virulence genes, novel sequence types, non-clinical, One Health

## Abstract

*Acinetobacter baumannii* is a Gram-negative, opportunistic pathogen that causes infections in the immunocompromised. With a high incidence of muti-drug resistance, carbapenem-resistant *A. baumannii* is designated as a priority 1 pathogen by the WHO. The current literature has expertly characterized clinical isolates of *A. baumannii*. As the challenge of these infections has recently been classified as a One Health issue, we set out to explore the diversity of isolates from human and non-clinical sources, such as agricultural surface water, urban streams, various effluents from wastewater treatment plants, and food (tank milk); and, importantly, these isolates came from a wide geographic distribution. Phylogenomic analysis considering almost 200 isolates showed that our diverse set is well-differentiated from the main international clones of *A. baumannii*. We discovered novel sequence types in both hospital and non-clinical settings and five strains that overexpress the resistance-nodulation-division efflux pump *adeIJK* without changes in susceptibility reflected by this overexpression. Furthermore, we detected a *bla*_ADC-79_ in a non-human isolate despite its sensitivity to all antibiotics. There was no significant differentiation between the virulence profiles of clinical and non-clinical isolates in the *Galleria mellonella* insect model of virulence, suggesting that virulence is neither dependent on geographic origin nor isolation source. The detection of antibiotic resistance and virulence genes in non-human strains suggests that these isolates may act as a genetic reservoir for clinical strains. This endorses the notion that in order to combat multi-drug-resistant infection caused by *A. baumannii,* a One Health approach is required, and a deeper understanding of non-clinical strains must be achieved.

**IMPORTANCE:**

The global crisis of antibiotic resistance is a silent one. More and more bacteria are becoming resistant to all antibiotics available for treatment, leaving no options remaining. This includes *Acinetobacter baumannii*. This Gram-negative, opportunistic pathogen shows a high frequency of multi-drug resistance, and many strains are resistant to the last-resort drugs carbapenem and colistin. Research has focused on strains of clinical origin, but there is a knowledge gap regarding virulence traits, particularly how *A. baumannii* became the notorious pathogen of today. Antibiotic resistance and virulence genes have been detected in strains from animals and environmental locations such as grass and soil. As such, *A. baumannii* is a One Health concern, which includes the health of humans, animals, and the environment. Thus, in order to truly combat the antibiotic resistance crisis, we need to understand the antibiotic resistance and virulence gene reservoirs of this pathogen under the One Health continuum.

## INTRODUCTION

*Acinetobacter baumannii* is a Gram-negative, hospital-acquired, opportunistic pathogen. It causes ventilator-associated pneumonia, urinary tract, and wound infections and can cause bacteremia in cases of treatment failure (1). Community-associated infections have been reported as rare but serious (2, 3). Additionally, carbapenem-resistant *A. baumannii* has been labeled a priority 1 pathogen by the World Health Organization (4) due to the inability to cure such infections. Due to the COVID-19 pandemic, the number of *A. baumannii* infections increased (5), and there is a high frequency of multi-drug resistance (MDR), upwards of 70% of strains (1). The presence of antibiotic resistance genes (ARGs) and virulence genes (VGs) is characteristic of *A. baumannii* genomes. The genome plasticity of *A. baumannii* is notable (6) as it is able to take up DNA readily from its surroundings, thus increasing the likelihood of MDR.

The clinical epidemiology of *A. baumannii* is being investigated globally using multi-locus sequence typing (MLST), outer core locus (OCL) typing, and capsule locus (KL) typing methodologies. MLST classifies isolates into clonal complexes based on allelic profiles of seven genes resulting in sequence type (ST) assignment (7), whereas OCL (8) and KL (9) typing use alleles of genes encoding the outer core assembly machinery and capsule production, respectively. Tracking of clonal spread also aids in tracking ARGs and VGs associated with particularly concerning clones. However, more and more often, *A. baumannii* from non-clinical sources (10–13) are being investigated because they too harbor ARGs and VGs. In fact, *A. baumannii* has been classified as a One Health problem (14), which encompasses all aspects of human, animal, and environmental health. Therefore, it is critical to study clinical as well as non-clinical isolates of *A. baumannii* from many different sources in addition to global distributions.

Herein, a collection of 36 isolates from hospital, tank milk, agricultural surface water, stream, and wastewater effluent (WWE) highlights the incredible diversity of *A. baumannii,* substantiates that ARGs and VGs are not unique to hospital isolates, and features similarities between clinical and non-clinical strains. Importantly, these isolates have been analyzed in the context of the main international clones described for *A. baumannii*, considering more than 149 additional genomes.

## RESULTS

### Novel sequence types exist within isolates from non-clinical and clinical sources

**Fig 1:**
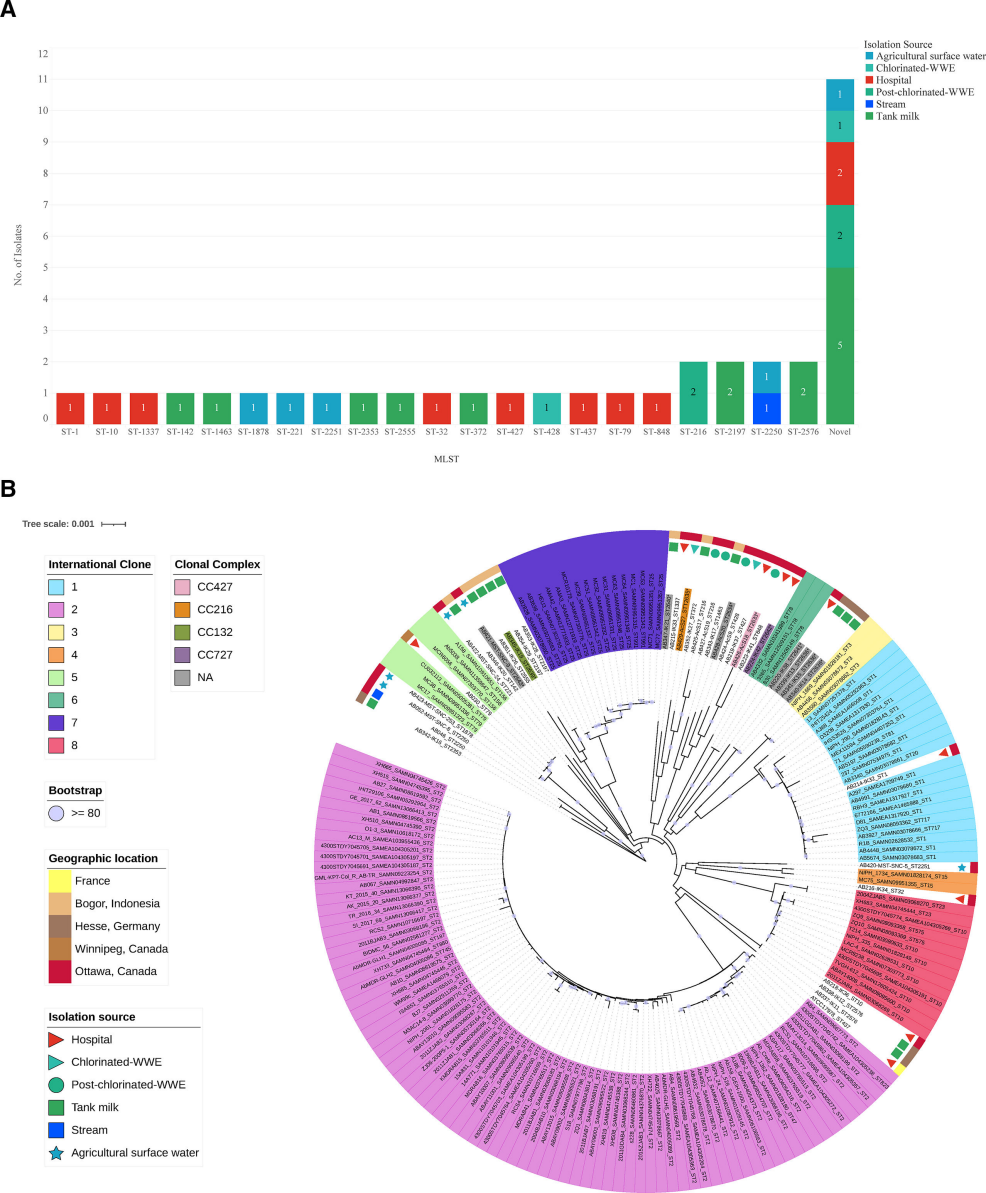
(A) Diversity of sequence types in clinical and non-clinical isolate collection. Each strain in the collection was typed based on the Pasteur method of sequence typing. The number of isolates that fit into each ST category is shown. Those that are of a novel ST are not necessarily the same ST, but they are grouped into a novel category together to show the count. The colors indicate the isolation source of each isolate. (**B**) Phylogenomic analysis of isolates. Representative whole genome sequences from all eight international clonal (IC) groups were analyzed alongside the whole genome sequences of this collection. IC groups are signified by the large rectangles in appropriate color surrounding the assembly name. Isolation source is shown by the small shapes on the second most outer circle. The geographic origin is shown on the outer circle. The clonal complex assignment is indicated in the coloured rectangles surrounding those of novel ST. Bootstrap values are indicated by a purple circle if they are >80. Those STs with asterisks indicate novel STs identified in this study. Color in the legend represents the isolation source, the geographic origin that corresponds to that of all figures, as well as clonal complex and IC assignment.

There is great genetic and phenotypic diversity within *A. baumannii,* and our collection of isolates showcases this. Many of these isolates were characterized as novel STs (Pasteur MLST typing scheme) (7). The majority of novel STs (*n* = 4, 44%) were recovered from tank milk samples (Fig. 1A) from two countries: Germany and Indonesia. Notably, there were two novel STs from hospital sources: AB220-IK38 and AB224-IK42. The other novel STs were isolated from chlorinated WWE (*n* = 1), post-chlorinated WWE (*n* = 2), agricultural runoff (*n* = 1), and hospital (*n* = 1) (Fig. 1A). The novel STs were assigned as follows: AB426-AcS18 (ST-2633), AB428-AcS20 (ST-2634), AB429-AcS27 (ST-2635), AB339-IK13, AB340-IK14, and AB341-IK15 as ST-2636, AB345-IK19 (ST-2639), AB347-IK21 (ST-2640), AB220-IK38 (ST-2641), AB224-IK42 (ST-2642), and AB421-MST-SNC-9 (ST-2643).

To further analyze this diversity, we examined the whole-genome sequences (WGS) using a phylogenomic approach and an additional 149 *A*. *baumannii* assemblies (Table S2). AB337-IK11 and AB338-IK12, (both ST-2576) isolated from tank milk (Hesse, Germany), form a new clade with the closest relation to the most prevalent IC2 and with ATCC17978 (ST-437) (Fig. 1B). The long length of the branch signifies this is a well-differentiated cluster and has strong clade support with bootstrap values above 80. Many of the human isolates clustered with IC1, IC5, IC6, and IC8. For example, one hospital isolate was classified as IC1 (AB214-IK32; ST1) and another as IC8 (AB218-IK36; ST10).

A highly diverse cluster exists in relation to IC7 (Fig. 1B) and is composed of six isolates. These isolates were recovered from a Canadian hospital, chlorinated and post-chlorinated WWE samples, and an Indonesian tank milk sample. The following STs make up this clade: ST-427, ST-428, ST-848, and ST-1463, and novel STs ST-2633 and ST-2634. There is a diverse array of clonal complexes (CCs) within this cluster as well. STs including ST-1463, ST-2634, ST428, and ST-2640 remain unassigned to any classified CC (Table S3). A novel ST, ST-2633, classified as CC427, is more closely related to AB223-IK41 (ST-848) than to AB219-IK37 (ST-427 and the founding member of CC427). Interestingly, another cluster including AB429-AcS27 (ST-2635 and CC216), from Canadian chlorinated-WWE, is grouped with Indonesian tank milk isolates, a Canadian hospital isolate, and with post-chlorinated-WWE isolates, AB425-AcS17 and AB427-AcS19 (ST-216). ST-216 is the founding member of CC216, which also includes AB429-AcS27. The detection of novel STs and unassigned CCs in the clinical, chlorinated, and post-chlorinated WWE and tank milk samples exemplifies the diversity of *A. baumannii,* as does the lack of IC2 detection in our collection of clinical isolates.

### Non-human isolates show reduced susceptibility to clinically relevant antibiotics and their genomes contain antibiotic-resistance genes

**Fig 2:**
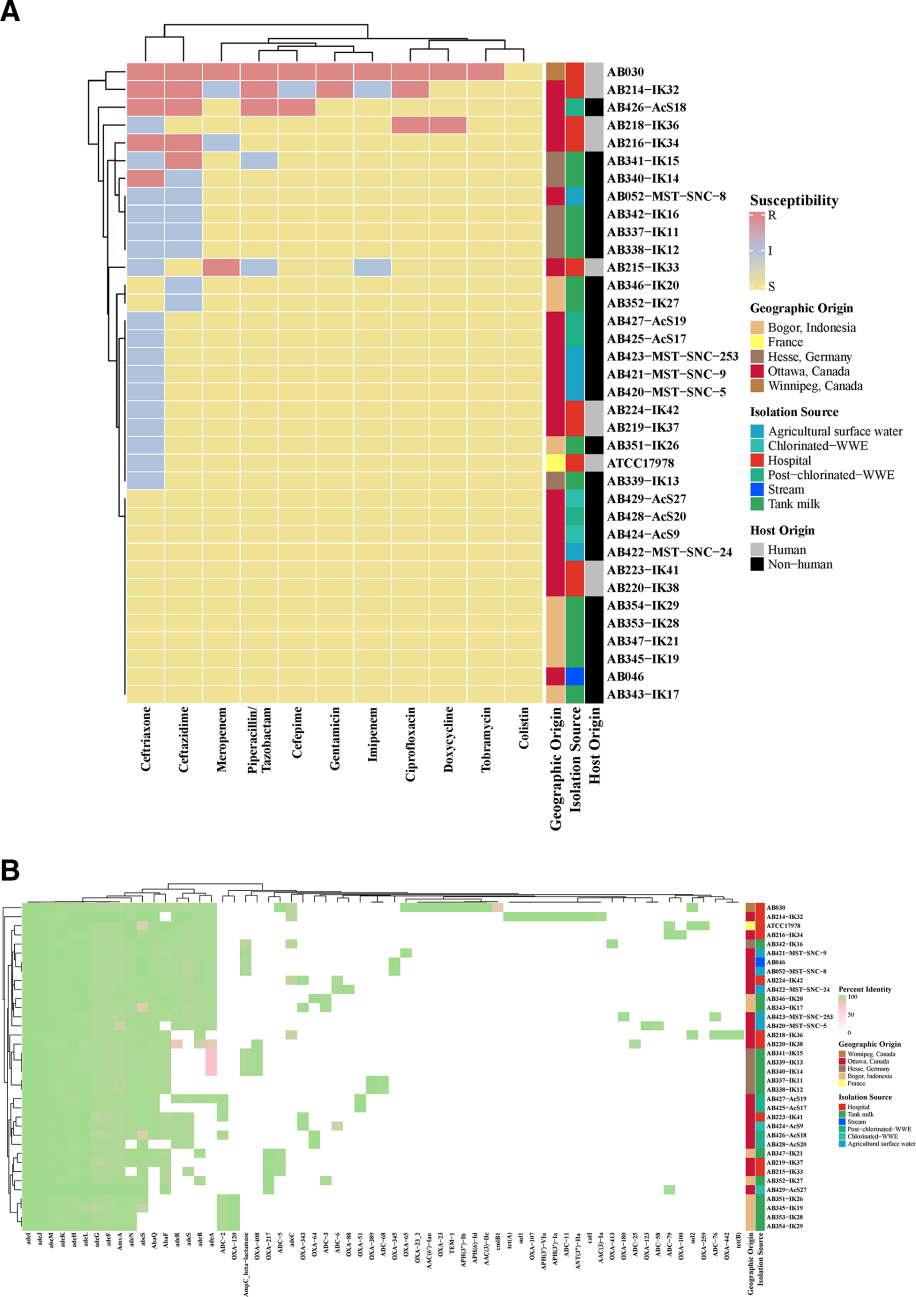
(A) Antibiotic susceptibility profiles of all strains. Susceptibility testing was performed according to the CLSI guidelines. Clinically relevant antibiotics tested are part of the CANWARD panel and are as follows: amikacin, cefazolin, cefepime, cefoxitin, ceftazidime, ceftobiprole, ceftolozane/tazobactam, ceftriaxone, ciprofloxacin, clarithromycin, clindamycin, colistin, daptomycin, doxycycline, ertapenem, gentamicin, imipenem, linezolid, meropenem, nitrofurantoin, piperacillin/tazobactam, tobramycin, trimethoprim/sulfamethoxazole, and vancomycin. Only those antibiotics that showed at least one strain with a change in susceptibility based on CLSI breakpoints are shown. Color in each panel represents the isolation source and the geographic origin that corresponds to that of all figures. (**B**) Antibiotic resistance gene profile of all isolates. Percent identity of each gene is used to explore the diversity. The minimum cutoff for nucleotide identity is 80% and is shown in pink. Hierarchical clustering demonstrates similarity based on the antibiotic-resistant gene profile. Color in each panel represents the isolation source and the geographic origin that corresponds to that of all figures.

Among this diverse set of isolates, antibiotic susceptibility profiles vary in terms of clinically relevant antibiotics. In this study, AB030 (15, 16) was used as a reference strain, in addition to ATCC17978. AB030 and AB214-IK32, both from hospitals, showed MDR phenotypes based on Clinical Laboratories Standards Institute (CLSI) breakpoints (Fig. 2A). All isolates are susceptible to colistin. AB426-AcS18 from post-chlorinated WWE shows decreased susceptibility to cephalosporins (ceftriaxone, ceftazidime, and cefepime) as well as the β-lactam antibiotic, piperacillin and its common β-lactamase inhibitor, tazobactam. The hospital isolate AB218-IK36 is resistant to doxycycline and ciprofloxacin. AB216-IK34 (hospital) displays resistance to ceftriaxone and ceftazidime. Another tank milk isolate, AB340-IK14, is resistant to ceftriaxone, and a clinical isolate, AB215-IK33, displays resistance to meropenem with intermediate susceptibility to imipenem, piperacillin/tazobactam, and ceftriaxone. Many of the non-human isolates (*n* = 17, 46%) showed intermediate resistance to ceftazidime, ceftriaxone, and piperacillin/tazobactam, all members of the β-lactam antibiotic family (Fig. 2A). A total of 7 human isolates displayed intermediate or resistant phenotypes to at least one antibiotic in comparison to 16 non-human strains.

The genetic contribution to antibiotic susceptibility was then investigated. AB030 showed the most numerous genetic resistance mechanisms. Cephalosporinase-encoding genes (*bla*_ADC-5_, *bla*_ADC-16_, and *bla*_ADC-18_) were scattered throughout the isolates, with *bla*_ADC-2_ being the most common (*n* = 8, 22%; Fig. 2B). One of the non-clinical strains, AB429-AcS27, was found to harbor *bla*_ADC-79_. Despite the presence of this gene, AB429-AcS27 is susceptible to all clinically relevant antibiotics tested (Fig. 2A). Finally, meropenem-resistant AB215-IK33 (hospital, Ottawa, Canada) only shows the presence of *bla*_OXA-217_ and *bla*_ADC-5_. Aminoglycoside-modifying enzyme-encoding genes were few: *aac (3)-Ia*, *ant(3″)-IIa*, *aph(3′)-VIa*, and *aph(3′)-Ia* were detected in MDR strain AB214-IK32, and *aac (3)-IIe*, *aph(3″)-Ib*, *aac (6)′-Ian,* and *aph (6)-Id* in MDR strain AB030 (Fig. 2B).

Efflux pump genes were highly conserved in all isolates. The genes encoding AmvA, AbeM, a member of the major facilitator superfamily, and small multidrug-resistant efflux pump family, respectively, were strictly conserved and found in all isolates. Conservation of the Rresistance-Nodulation-Division (RND) family of efflux pumps is not a unique feature of *A. baumannii* (17), and these isolates are consistent with previous studies. Efflux operons *adeFGH* and *adeIJK* are found in all isolates, regardless of their geographic origin or isolation source. *adeAB* is the least conserved RND pump, with 49% (*n* = 18) of isolates encoding either *adeA* or *adeB*. The corresponding outer membrane factor, *adeC*, however, is much less conserved and is found only in 14% (*n* = 5) of isolates.

### Variation in efflux pump expression cannot only be explained by variation in promoter regions or regulators

**Fig 3:**
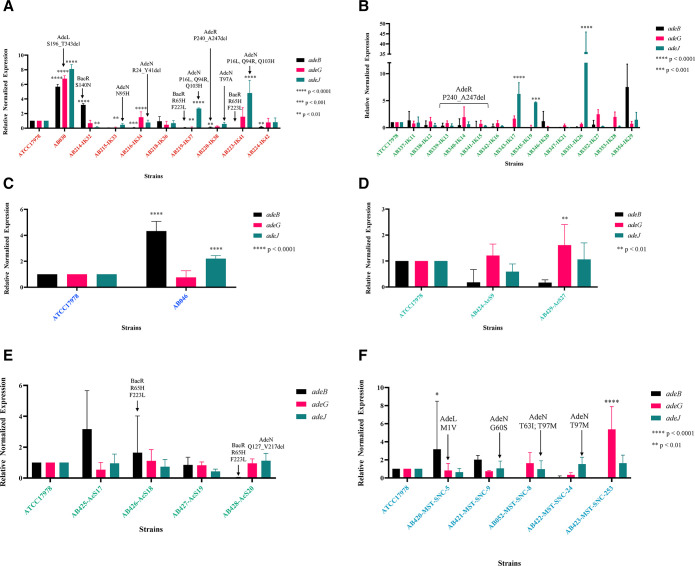
Resistance-nodulation-division efflux pump expression of all strains. Expression was performed using total RNA converted to cDNA (see Materials and Methods for more details) using the primers listed in Table S1. The Pfaffl method was used to calculate the relative fold change in expression compared to the type-strain ATCC17978. Two-way ANOVA was used in GraphPad Prism 10.0.0 to determine significance. Significance values are indicated in each panel. Color in each panel represents the isolation source that corresponds to that of all figures. (**A**) Isolates from clinical source. (**B**) Isolates from tank milk source. (**C**) Isolates from stream source. (**D**) Isolates from post-chlorinated WWE. (**E**) Isolates from chlorinated WWE. (**F**) Isolates from agricultural surface water.

Investigation into the expression of RND pumps in this collection of isolates was performed using the second gene in each operon as a proxy for expression. Within the clinical strains, AB030 displayed significant upregulation of all three characterized RND efflux pumps, while *adeIJK* was upregulated in two susceptible clinical isolates, AB219-IK37 and AB223-IK41 (Fig. 3A).

Overexpression of *adeIJK* was also observed in two isolates from tank milk, AB343-IK17 and AB351-IK26, as well as a stream isolate, AB046 (Fig. 3B and C). The promoter region of *adeIJK* was investigated to determine possible explanations for this overexpression. It was 100% conserved between all isolates (data not shown), including the operator for AdeN, the TetR-type regulator that exerts transcriptional control over *adeIJK* (18). Additional analysis was done for AdeN as well. Each isolate had 100% identity to the query sequence of AdeN, WP_000543879.1, except AB052-MST-SNC-8, AB428-AcS20, AB422-MST-SNC-24, AB220-IK38, AB215-IK33, AB421-MST-SNC-9, AB216-IK34, AB219-IK37, and AB223-IK41(*n* = 9; 25%). None of these mutations affected the helix-turn-helix DNA-binding domain of these AdeN variants (data not shown), but most are found in the dimerization domain, which is critical for AdeN function (Fig. S1a). AdeN of AB052-MST-SNC-8 contains T63I and T97M. AB428-AcS20 appears to have a truncation of AdeN at Q127_V217del (noted in Fig. 3 and in Fig. S1). AB422-MST-SNC-24 shares the same T97M as AB052-MST-SNC-8, while AdeN of AB220-IK38 shows T97A. There is an N95H mutation in AB215-IK33 and a G60S mutation in AB421-MST-SNC-9 (Fig. S1a). In strain AB216-IK34, there is a deletion of 17 amino acids (R24_Y41del) and both AB219-IK37 and AB223-IK41 (hospital, Ottawa, Canada) have P16L, Q94R and Q103H mutations (Fig. S1b).

AB214-IK32 showed overexpression of *adeABC* (Fig. 3A), which may contribute to its MDR phenotype (Fig. 2A). The stream isolate AB046 also showed *adeAB* overexpression as did AB420-MST-SNC-5 (Canadian agricultural surface water) (Fig. 3C and F). Hospital isolates AB216-IK34 and AB220-IK38 had a downregulation of *adeAB*. Of those isolates showing the presence of *adeAB(C*), there was 100% nucleotide identity (data not shown). To investigate if the expression difference of AB214-IK32, AB220-IK38, and AB046 was due to possible regulator mutations, the two-component systems, AdeRS and BaeSR were investigated.

No mutations appeared in AdeR of AB214-IK32, AB046, and AB420-MST-SNC-5. Further analysis of all isolates showed 100% conservation in the receiver domain, known to interact with its cognate sensor histidine kinase, AdeS (data not shown). However, the DNA-binding domain of AdeR in AB220-IK38, AB339-IK13, AB340-IK14, and AB341-IK15 was truncated by seven amino acids (Fig. S2a) while showing 100% identity to each other (Fig. S2b). Furthermore, investigation into BaeR revealed a common mutation (R65H) observed in AB219-IK37, AB223-IK41 (both hospital strains, Ottawa, Canada), and AB426-AcS18 (post-chlorinated WWE, Ottawa, Canada). AB214-IK32 from a Canadian hospital shows an S140N. Finally, a last group of strains displayed the F223L mutation including AB219-IK37, AB223-IK41 (hospital, Ottawa, Canada), AB426-AcS18, AB428-AcS20 (post-chlorinated WWE, Ottawa, Canada), and AB353-IK28 and AB354-IK29 (tank milk, Bogor, Indonesia). The impact of these mutations is unknown and requires further study.

Regarding *adeFGH* expression, AB030 (hospital, Winnipeg, Canada) showed overexpression (Fig. 3A) in agreement with previous studies (16), as did AB423-MST-SNC-253 (agricultural surface water, Ottawa, Canada) (Fig. 3F) and AB429-AcS27 (chlorinated WWE, Ottawa, Canada) (Fig. 3E). The promoter region of *adeFGH,* which includes a portion of the reverse complement of its known regulator, *adeL*, as well as the AdeL regulatory site, showed 100% conservation for all isolates (data not shown). In AB030, however, there was a deletion of AdeL from S196 to the terminus at T343 (Fig. S3). As AdeL is a known repressor of *adeFGH*, this may provide an explanation as to why *adeG* is overexpressed in this isolate. Additionally, only one other isolate demonstrated variation in AdeL: AB420-MST-SNC-5 and mutation has an unknown impact on AdeL function (Fig. S3).

### Virulence is not associated with isolation source or geographic origin

**Fig 4:**
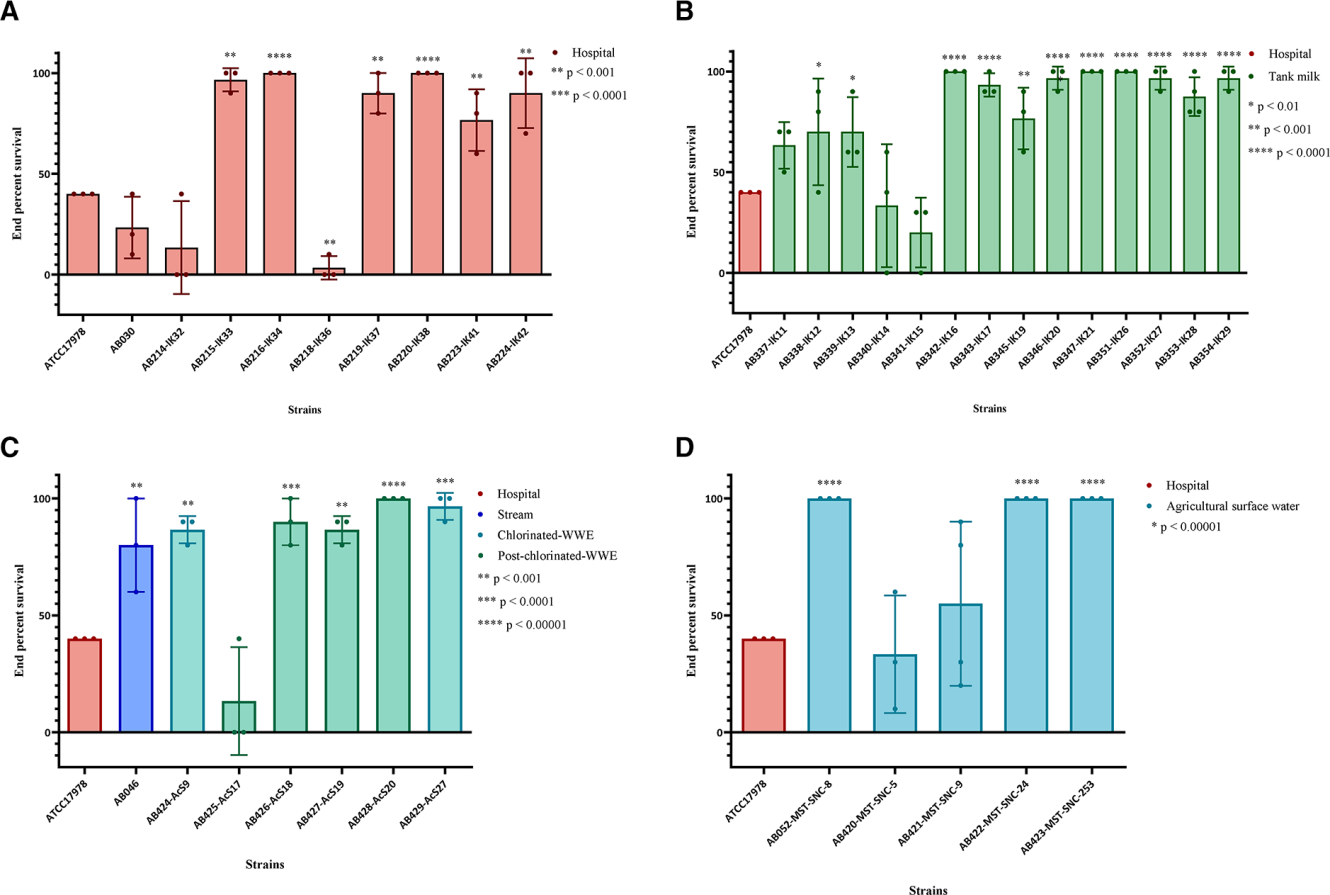
Virulence of hospital isolates in *Galleria mellonella*. Larvae were selected, wiped with 70% ethanol using a sterile swab, and then injected in the back left proleg. Survival was measured every 12 hours for a total of 72 hours. Each biological replicate is represented as one point for each strain and is composed of 10 biological replicates for a total of at least 30 larvae for each strain. Statistics were performed in GraphPad Prism 10.0.0. One-way ANOVA was performed to determine significance. *P < 0.1, **P < 0.01, ***P < 0.001, and ****P ≤ 0.0001. Color in each panel represents the isolation source that corresponds to that of all figures. (**A**) Virulence of hospital isolates from Ottawa, Canada. (**B**) Virulence of tank milk isolates from Bogor, Indonesia and Hesse, Germany. ATCC17978 is included for comparison despite being a hospital isolate and is therefore indicated in red. (**C**) Virulence of the stream isolate, the chlorinated WWE, and post-chlorinated WWE isolates, all from Ottawa, Canada. ATCC17978 is included for comparison despite being a hospital isolate and is therefore indicated in red. (**D**) Virulence of agricultural surface water isolates. ATCC17978 is included for comparison despite being a hospital isolate and is therefore indicated in red.

Virulence is an important characteristic of pathogens and *A. baumannii* is no exception. The wax moth larvae insect model, *Galleria mellonella*, is a valid model to evaluate virulence in *A. baumannii* (19). Relative to ATCC17978, clinical isolates AB215-IK33, AB216-IK34, AB219-IK37, AB220-IK38, and AB224-IK42 (*n* = 5, 41% of clinical isolates) showed higher end point percent survival, indicating less virulence (Fig. 4A). AB218-IK36 was the only isolate showing higher virulence than ATCC17978. The remaining clinical isolates showed no significant difference and therefore were equally as virulent as ATCC17978. German tank milk isolate AB342-IK16 and all Indonesian tank milk isolates showed higher survival rates (Fig. 4B). AB046 from a Canadian stream, Canadian chlorinated WWE isolates (*n* = 2) and three isolates from Canadian post-chlorinated WWE (Fig. 4C), as well as AB052-MST-SNC-8, AB422-MST-SNC-24 and AB423-MST-SNC-253 from Canadian agricultural surface water (Fig. 4D), showed less virulence.

**Fig 5:**
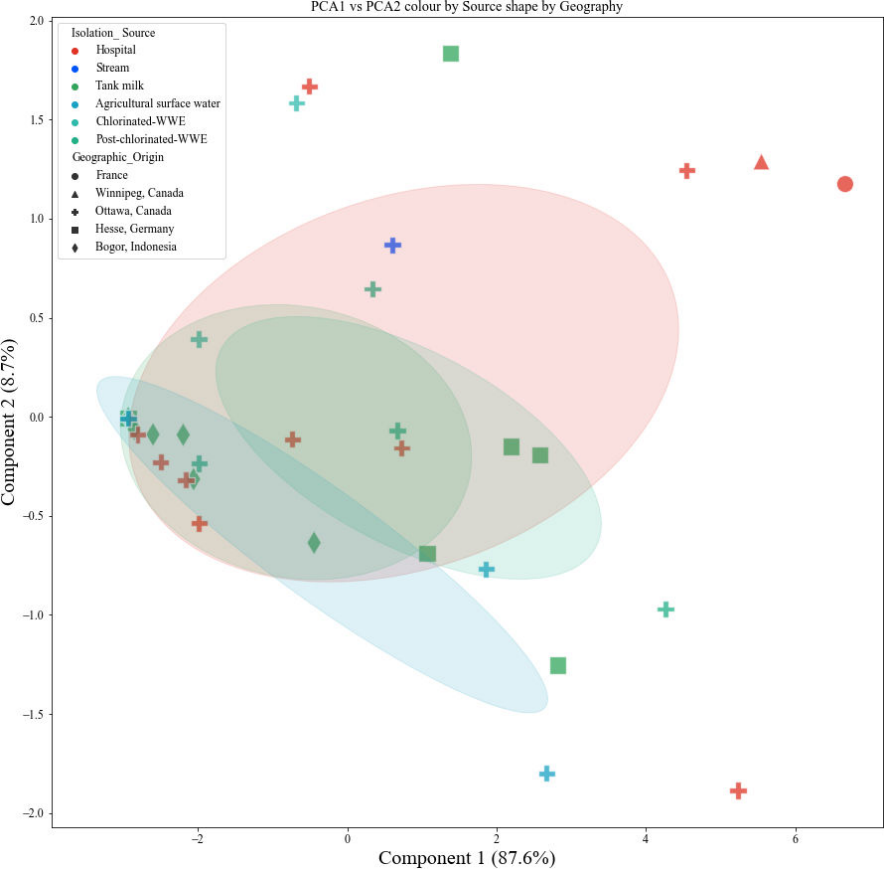
Principal component analysis of survival of *G. mellonella*. Survival data were used to determine if clinical and non-clinical isolates clustered together. Isolation source is indicated in various colors, and geographic origin is noted as varying shapes. Ellipses include an area that is 1 standard deviation from the mean with 95% confidence intervals. Ellipses’ colors match the isolation source color that corresponds to the cluster.

Hospital isolates from Ottawa, Canada clustered together with tank milk isolates from Bogor, Indonesia and post-chlorinated WWE isolates from Ottawa, Canada (blue ellipsis, Fig. 5) based on principle component analysis (PCA). These isolates have similar virulence profiles, suggesting virulence is independent of geographic origin and isolation source. Furthermore, in a separate cluster, German tank milk strains grouped with clinical isolates from Ottawa, Canada (green ellipsis, Fig. 5). There are also those strains whose virulence profiles are distinct from any other strain in the collection, for example, the type-strain ATCC17978 (red circle, top right, Fig. 5). Clusters of clinical and non-clinical strains highlight overlap in virulence between these strains from widely different geographic origins and isolation sources.

### The presence or absence of virulence genes does not explain virulence profiles in *G. mellonella*

**Fig 6:**
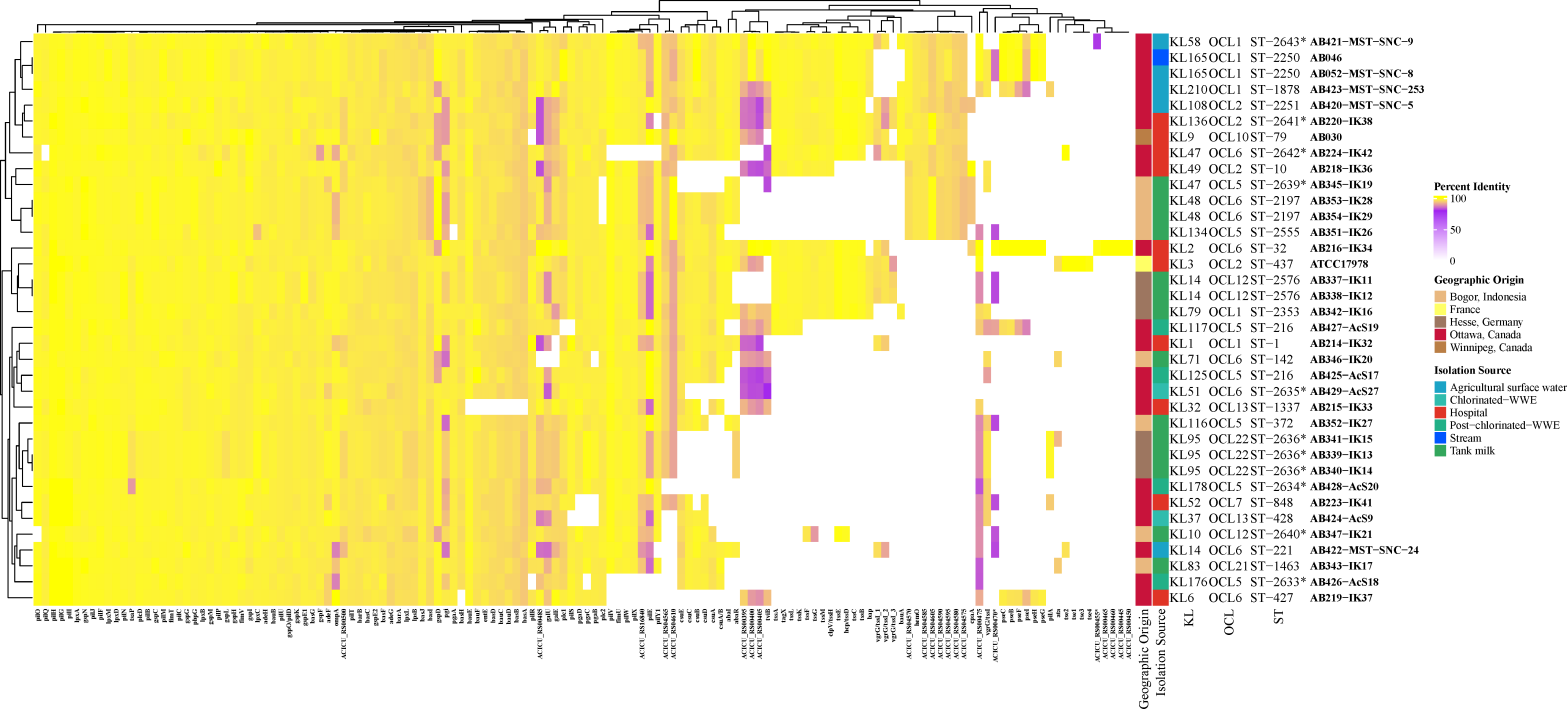
Virulome determination of all isolates. Percent identity of each gene is used to compare the diversity. The minimum cutoff for nucleotide identity is 80% and is shown in purple. Hierarchical clustering demonstrates similarity based on the virulence gene profile. Capsule locus, outer core locus, and sequence type are listed for each isolate. Those STs with asterisks indicate novel STs identified in this study. Color in each panel represents the isolation source and geographic origin that corresponds to that of all figures.

In order to understand the genetic components behind the virulence profiles observed, the VGs of all strains were investigated. Non-clinical and clinical isolates, regardless of geographic origin, cluster together as there are similarities in VG profiles (Fig. 6).

The outer-membrane-encoding gene, *ompA*, was conserved in all isolates (Fig. 6). The glycoprotease gene, *cpaA*, was detected in hospital (AB216-IK34 and AB218-IK36), tank milk (AB346-IK20 and AB354-IK29), and agricultural surface water (AB420-MST-SNC-5, AB421-MST-SNC-9, and AB422-MST-SNC-24) isolates. There was also a high conservation of the *pil* genes involved in the production, secretion, and assembly of the type 4 pili (T4P) used in surface attachment and twitching motility. The most significant variation was observed in *pilE,* which encodes a minor pilin subunit and has not been well characterized in *A. baumannii*. Furthermore, only AB224-IK42, AB339-IK13, AB340-IK14, AB341-IK15, AB342-IK16, and AB423-MST-SNC-253 have alleles of the major pilin subunit, *pilA,* above 80% identity. Interestingly, ATCC17978 had the highest motility and appeared to be associated with forming lower amounts of biofilm (Fig. S4a through d and S5a through d). This was interrogated further in all isolates via (Spearman’s) correlation analysis that was not significant (*R* = −0.1382, *P* = 0.4016); thus, no correlation between biofilm and motility was observed (Fig. S6).

Typing and epidemiological tracking of *A. baumannii* and other Gram-negative pathogens not only consist of MLST but also of OCL and KL typing. OCL and KL typing also provide a starting point for determining lipopolysaccharide and capsular polysaccharide (CPS) structures. The majority of these isolates were typed as OCL6 (*n* = 8, 22%), including AB224-IK42 (hospital). In our collection, associated STs with OCL6 are ST32, ST142, ST134, ST221, ST427, and three novel STs. Six (17%) isolates are OCL1, including isolates from clinical and non-clinical sources. The OCL2 designation was assigned to three isolates, including the type-strain ATCC17978, AB420-MST-SNC-5 from chlorinated WWE, and AB220-IK38 from hospital. A wide distribution of KL types was observed. This includes the most common KL14 and KL95 (each *n* = 3, 8%). Those KL types associated with at least one isolate of a novel ST include KL14, KL10, KL47, KL48, KL58, KL79, KL83, KL95, KL108, KL116, KL134, KL136, KL165, KL176, KL178, and KL210 (Fig. 6). The most common clinical KL type is KL9 (20), and only AB030 (hospital, Canada) was identified as KL9, further supporting the unexplored diversity of *A. baumannii*.

## DISCUSSION

Overall, the extensive diversity of *A. baumannii*, both genetic and phenotypic, is highlighted in this study. This collection encompasses a distinct set of human and non-human isolates from diverse isolation sources and geographic origins. The detection of novel STs from clinical and non-clinical sources exemplifies this non-uniformity. Despite IC2 being the dominant IC in Canada (21), none of these hospital isolates were categorized as such. As shown in Fig. 1B, one hospital isolate, AB214-IK32, was classified as an IC1, which is the second most prevalent Canadian clonal complex. AB218-IK36 (ST10) is IC8, which was also previously detected in Canada (21). The lack of relation between the clinical isolates and those already characterized in Canada suggests clonal replacement of other lineages, at least in Ottawa, Canada, since the most recent study in 2019 (21).

AB337-IK11 and AB338-IK12 (tank milk, Germany) are unique because they cluster between IC1 and IC8 and form their own well-differentiated clade (Fig. 1B). WGS phylogenetic characterization indicates that they belong to the same ST and CC, albeit novel. CC determination using the Pasteur MLST was elucidated by Gaiarsa et al. (22) and involves evaluating relationships between all known STs and discovering the founder ST. By using this method, the understanding of the spread and the epidemiological tracking of *A. baumannii* can be completed on a global scale, and our study adds to this area of research. AB337-IK11 and AB338-IK12 (tank milk, Germany) are interesting due to their KL and OCL types; both strains are KL14 and OCL12 (Fig. 6). This collection contains isolates of the most recently identified OCL types—OCL17–22 (23): one isolate from Indonesian tank milk, AB343-IK17 (OCL21) and three from OCL22 namely AB341-IK15, AB339-IK13, and AB340-IK14, all from German tank milk and all with novel STs (Fig. 6). ST32 has previously been associated with OCL6 (23), whereas our study shows that ST142, ST134, ST221, ST427, and three novel STs have now been associated with this OCL type. AB337-IK11 and AB338-IK12 also reveal intriguing hierarchical clustering based on VG analysis. Not only do they cluster together but also cluster with clinical isolate ATCC17978 (Fig. 6), as they did in the phylogenomic analysis (Fig. 1B), and both do not show a significantly different virulence profile compared to ATCC17978 (Fig. 4B). These two isolates highlight the diversity of non-human strains. The distribution of hospital isolates throughout the diverse clade near IC5 also exemplifies these dissimilarities between WGS of clinical and non-clinical strains (Fig. 1B).

This collection of *A. baumannii* shows great variation in antibiotic susceptibility, ARG profile, virulence in *G. mellonella,* and VG profile. Hierarchical clustering suggests that there is no clear-cut trend with regard to susceptibility and isolation source or geographic origin (Fig. 2A). The genes *bla*_ADC-79_*, bla*_ADC*-*3_, and *bla*_OXA-217_ were detected in AB429-AcS27 (Canadian chlorinated WWE). Tight control of β-lactamase expression is maintained and only induced in the presence of the β-lactam drug (24), suggesting that the presence of the antibiotic alone should induce expression and therefore confer resistance. However, AB429-AcS27 is susceptible to all antibiotics tested, including all β-lactam antibiotics, signifying alternate expression control in this strain. Another strain that showed the presence of *bla*_OXA-217_ was AB215-IK33 (Canadian hospital). In contrast to AB429-AcS27, this isolate is resistant to meropenem and upon further investigation, both *bla*_ADC-5_ and *bla*_OXA-217_ genes were detected. The *bla*_OXA-217_ is a *bla*_OXA-51-like_ β-lactamase gene and although not specifically characterized, members of this group are known to hydrolyze imipenem and meropenem (25). Therefore, it is likely responsible for this phenotype in AB215-IK33. Insertional elements, such as IS*Aba*1, are known to increase the expression of *bla* genes such as *bla*_OXA-23-like_, *bla*_OXA-51-like_, *bla*_OXA-58-like_, *bla*_OXA-235-like_ genes (25), and *bla*_ADC-79_ (26). Further work determining the expression control of *bla*_OXA-217_ in AB215-IK33 (resistant to meropenem) and AB429-AcS27 (sensitive to meropenem and other antibiotics tested) as well as *bla _ADC-79_* in AB429-AcS27 will be critical to understanding these phenotypes in non-clinical isolates. These two strains, among others, exemplify the crucial need to test susceptibility in addition to investigation of the ARGs. Additionally, the detection of *bla* in non-clinical *A. baumannii* is not unique to this study (11, 27, 28). However, the detection of these elements in *A. baumannii* from non-clinical sources, such as water and soil, suggests that they act as reservoirs for ARGs, especially β-lactamases (25) and this analysis supports this.

Overexpression of RND efflux pumps is a major contributor to MDR in clinical *A. baumannii* (29–31); however, these pumps are not always expressed. Five strains showed overexpression of *adeIJK* without presenting an MDR phenotype (Fig. 3). Identified substrates of AdeIJK include β-lactams, chloramphenicol, tetracycline, erythromycin, lincosamides, fluoroquinolones, fusidic acid, novobiocin, rifampin, and trimethoprim (32). AdeIJK is known as the universal *Acinetobacter* spp. efflux pump and is typically constitutively expressed (33); therefore, this overexpression is surprising. AB219-IK37 (hospital, Canada) and AB351-IK26 (tank milk, Indonesia) are intermediately resistant to ceftriaxone, while AB223-IK41 (hospital, Canada), AB343-IK17 (tank milk, Indonesia), and AB046 (stream, Canada) are susceptible to all antibiotics tested, including those substrates of AdeIJK tested. The analysis of AdeN of AB052-MST-SNC-8, AB422-MST-SNC-24, AB421-MST-SNC-9 (all agricultural surface water, Canada), AB428-AcS20 (post-chlorinated WWE, Canada), AB215-IK33, AB219-IK37, AB220-IK38, and AB223-IK41 (all hospital strains, Canada) revealed a common variance at T97 (*n* = 2: T97M and *n* = 1: T97A), as well as at P16L, Q94R, and Q103H (*n* = 2), N95H, G60S, T63I, and a truncation at Q127_V217del (Fig. 3) all located in the dimerization domain (Fig. S1). Dimerization of TetR-type repressors, including AdeN, is essential for function. However, none of these mutations affect the helix-turn-helix DNA-binding domain of AdeN. The P16L, Q94R, and Q103H mutations in both AB219-IK37 and AB223-IK41 could explain the overexpression of *adeIJK* in these strains. This does not, however, explain the susceptibility of these strains to those substrates of AdeIJK (Fig. 2A). Considering there is no overexpression of *adeIJK* in any of the other strains with AdeN mutations (Fig. 3), this needs further investigation with additional sequencing and characterization. The truncation of AdeR in AB220-IK38 (hospital, Ottawa, Canada) may be attributed to the decreased expression of *adeB,* but further experimentation is required.

As antibiotic selective pressure is higher in clinical settings, we predicted that those isolates from clinical isolation sources would display higher levels of resistance. However, this was not the case. Two hospital isolates, AB220-IK38 and AB223-IK41, were susceptible to all antibiotics tested and clustered with those of non-clinical origin (Fig. 2A). Vice versa, AB426-AcS18 (post-chlorinated WWE) clustered with hospital isolates. In almost all cases, hierarchical clustering created different groupings based on ARG and antibiotic susceptibility profiles. It is notable that AB030 and AB214-IK32 clustered together (Fig. 2A) as having the most similar susceptibility profiles, as well as the most similar ARG profile (Fig. 2B). This is also despite being from different ICs (AB030: IC5 and AB214-IK32: IC1) and different geographic origins (AB030: Winnipeg, Canada and AB214-IK32: Ottawa, Canada). A susceptibility-based cluster includes AB052-MST-SNC-8 (Canadian agricultural surface water), AB342-IK16 (German tank milk), AB337-IK11 (German tank milk), and AB338-IK12 (German tank milk) (Fig. 2A), whereas AB052-MST-SNC-8 clusters with AB224-IK42 (Canadian hospital) in the ARG analysis (Fig. 2B). A hospital isolate and an agricultural surface water isolate clustering together due to similar ARG profiles but clustering separately based on susceptibility phenotypes is an intriguing finding. One shortcoming of this study is that expression for every ARG was not determined, and this may explain some of the differential clustering between ARG and susceptibility profiles. However, these data provide additional support for the need for phenotypic testing of resistance instead of genetic analysis alone.

There is a bias in the literature toward virulence characterization using an animal model for only those isolates from hospitals (34, 35). To our knowledge, only two non-clinical isolates have been investigated for virulence (10, 36). Therefore, we investigated if the virulence profiles of our hospital isolates were comparable to those from non-clinical sources. The majority of the clinical isolates were less virulent compared to ATCC17978 (Fig. 4). This collection of isolates represents strains from non-dominant ICs, such as IC1, IC4, IC7, and IC8, which broadens our understanding of the diversity of *A. baumannii* virulence. Interestingly, the clinical isolates were undifferentiated from those of non-clinical sources (Fig. 5). The lack of delineation between clinical and non-clinical isolates suggests that the isolation source is not a good indicator of virulence. It also suggests that a non-human isolate of *A. baumannii* could just as easily become a pathogen if given the opportune conditions.

It is intriguing that a high variation in *pilA* is observed. PilA is the major subunit of the T4P, and there is evidence of convergent evolution with other bacterial species (37). Modification of pilins via glycosylation supports host immune evasion (38, 39) and in a similar fashion, variation or lack of canonical *pilA* may also provide immune avoidance strategies. Furthermore, in other studies, there appears to be an inverse correlation between biofilm formation and motility (40) in a PilA-dependent manner (37). However, our data do not corroborate this finding (Fig. S6). When performing a Spearman correlation analysis between motility and biofilm biomass, we did not find a correlation (*R* = −0.1382, *P* = 0.4016). This may be due to the fact that our study included non-human *A. baumannii,* where previous work was done in clinical isolates.

It is interesting that *cpaA* was detected in non-clinical isolates. CpaA has been characterized in clinical isolates of *Acinetobacter* spp. as a key player in the hydrolysis of human *O*-linked glycoproteins whose cleavage results in blood coagulation (41, 42) and complement disruption (43). To our knowledge, the characterization of such a potent virulence factor has been limited to clinical isolates, and therefore this presents an interesting avenue for further study. As a potent glycoprotease, only known to cleave human *O-*linked glycoproteins, the conservation of *cpaA* in those isolates from the non-clinical origins suggests alternate functions or conservation of surrounding genetic elements that are advantageous. Furthermore, this also highlights the pathogenic potential of *A. baumannii* from non-clinical sources.

Many non-human bacterial isolates harbor ARGs as a means to protect themselves from other antibiotic producers in their environmental communities (44). To truly understand the origin of antibiotic resistance and virulence traits in *A. baumannii,* isolates from all environments must be characterized, as well as the microbial communities surrounding them, including antibiotic producers and their genes (45). Studies have shown that ARGs identified in soil-dwelling microbes have been mobilized to clinical isolates (46).

In conclusion, these results show that by exploring the diversity of *A. baumannii* genetically and phenotypically, the prevalence of ARGs in non-clinical isolates becomes even more apparent and that these non-clinical isolates act as reservoirs for their clinical counterparts. Very few *bona fide* VGs are attributed to *A. baumannii,* where mechanisms to resist desiccation, withstand harsh conditions, and evade host immune defenses are hallmarks of successful pathogenic strains (38, 39). We also demonstrate the high variability of VGs, requiring investigation for the development of vaccines and classification of novel VGs and alleles. Our study greatly contributes to the variation and wide distribution of ARGs and VGs in *A. baumannii* especially in non-human isolates. Finally, in order to combat the ever-evolving infections caused by *A. baumannii*, a One Health approach must be taken. Non-clinical strains harboring ARGs and VGs may be an important genetic reservoir for clinical isolates and represent the unexplored diversity of *A. baumannii*.

## MATERIALS AND METHODS

### Strain isolation

A summary of the strains isolated for this study can be found in Table S1.

### Whole-genome sequencing

Detailed methods for the extraction of DNA and sequencing can be found in Supplemental Methods. All assemblies have been deposited to NCBI GenBank under the BioProject number PRJNA819071 and BioSample numbers SAMN26898552–SAMN26898587.

### Species determination, phylogenomic analysis, sequence typing, outer core locus typing, and capsule typing

The average nucleotide identity was calculated with pyani v.0.2.9 (47) using Mummer 3.0 (48) to confirm the species using the *A. baumannii* strain ATCC 19606 (BioSample: SAMN13045090). Only isolates above 95% of identity with the reference strain were considered for further analysis. The phylogenomic analysis was performed as previously described (49). In brief, a pangenome analysis was run by means of Roary (50) using 95% identity as the cutoff value. The genes in a single copy in all the genomes, commonly known as single gene families (SGFs), were recovered from the pangenome matrix. Then, the SGFs were aligned and tested for recombination. A maximum likelihood phylogeny was built with 471 SGF without recombination, and finally, the tree was annotated. The assemblies used can be found in Table S2. Sequence typing was performed using the Pasteur scheme (7) as a reference via ABRicate (51). Outer core locus and capsule typing were performed using Kaptive (9).

### MIC determination

Antibiotic susceptibility was evaluated as per the CLSI guidelines using broth microdilution methods. The full CANWARD panel (52) of antibiotics was used for testing. Based on CLSI breakpoints, isolates were categorized into susceptible (S), intermediate (I), and resistant (R) (53). MIC values can be found in Table S4. The data were displayed using pheatmap in R Studio.

### Antibiotic resistance gene determination

The ABRicate pipeline (51) was used and directed to use the Comprehensive Antibiotic Resistance Database (54) for the analysis of all genomes with perfect (100% identity) and strict (≥80% identity) hit coverage of 80% or greater. Output was then displayed using the ComplexHeatmap package (55) in R Studio.

### RND efflux expression evaluation

Overnight cultures of each strain were subcultured 1/100 in LB broth with shaking at 37°C until the mid-exponential phase (*A*_600_ of 0.7 ± 0.5). The cells were pelleted, and the supernatant was removed before storing overnight at −70°C. RNA was extracted using the Invitrogen Purelink RNA extraction kit (Thermo Fisher, Waltham, USA), according to the manufacturer’s protocol. Then, 1 µg of eluted RNA was treated with 2 U of DNase using the Invitrogen Purelink DNase kit (Thermo Fisher, Waltham, USA) and incubated at 37°C for 30 minutes, followed by inactivation at 62°C for 5 minutes. cDNA was synthesized using the Invitrogen VILO cDNA kit (Thermo Fisher, Waltham, USA) from 1 µg DNase-treated RNA. Incubation conditions were as follows: 25°C for 10 minutes, 42°C for 60 minutes, and then 85°C for 5 minutes. The newly synthesized cDNA was diluted 1/5 prior to analysis using RT-qPCR. Applied Biosciences (Beverly Hills, USA) SYBR Select Master Mix was combined with appropriate volume of sterile mQH_2_O and primers to a final concentration of 200 nM. Primer sequences can be found in Table S5. Diluted cDNA template was added so that 200 ng of DNA is in each reaction. The reaction was run on an Applied Biosciences (Beverly Hills, USA) StepOnePlus system with the following thermal cycling parameters: 50°C for 2 minutes, 95°C for 2 minutes, then 40 cycles of 95°C for 15 seconds, 60°C for 1 minute, with a final melt curve stage of 95°C for 10 seconds, 60°C for 5 seconds, and a step up to 95°C for 10 seconds increasing temperature by 0.3°C. RT-qPCR for each strain was performed with three technical replicates and at least three biological replicates per target gene. The Pfaffl method of analysis was used to calculate the relative fold change expression compared to ATCC17978. One-way ANOVA was performed using GraphPad Prism 10.0.0.

### Virulence gene determination

The ABRicate pipeline (51) was directed to use the Virulence Finder Database (56) to analyze all genomes with ≥80% nucleotide identity minimum and hit coverage of ≥80%. Output was then displayed using the ComplexHeatmap package (55) in R Studio.

### Survival assays with *Galleria mellonella*

Upon arrival, larvae were sorted based on size and all those that were deemed equal were used. Larvae were injected within 1 week of arrival on site. Overnight cultures of *A. baumannii* were standardized to 0.5 MacFarland and then diluted 1/100 in 0.85% sterile saline. The ventral side of each larva was swabbed with 70% ethanol in preparation for injection. Then, 10 µL of standardized diluted culture was injected into the left hind proleg. The larvae were then incubated in sterile plastic petri plates at 37°C for 72 hours. Survival was measured every 12 hours. At the beginning of each injection session, sterile 1× PBS was injected into 10 larvae, as well as at the end to ensure syringe washes were adequate. A total of 10 technical replicates and at least 3 biological replicates were performed for each strain. Percent end-point survival was calculated, and one-way ANOVA in GraphPad Prism 10.0.0 was used to evaluate significance compared to ATCC17978. PCA was performed in Python using Pandas and visualized using Seaborn and Matplotlib.

## Data Availability

NCBI GenBank accession numbers: BioProject PRJNA819071 and BioSample SAMN26898552–SAMN26898587.

## SUPPLEMENTAL MATERIAL

Supplemental Figures msphere.00741-23-s0001.pdf Figures S1-S6.

Supplemental Text msphere.00741-23-s0002.docx Supplemental methods and figure legends.

Supplemental Tables msphere.00741-23-s0003.docx Tables S1, S3, and S5.

Table S2 msphere.00741-23-s0004.xlsx Metadata for assemblies used for phylogenomics analysis.

Table S4 msphere.00741-23-s0005.xls MIC values.

ASM does not own the copyrights to Supplemental Material that may be linked to, or accessed through, an article. The authors have granted ASM a non-exclusive, world-wide license to publish the Supplemental Material files. Please contact the corresponding author directly for reuse.

## References

[1] Antunes LCS, Visca P, Towner KJ. 2014. Acinetobacter baumannii: evolution of a global pathogen. Pathog Dis 71:292–301. doi:10.1111/2049-632X.12125 24376225

[2] Dexter C, Murray GL, Paulsen IT, Peleg AY. 2015. Community-acquired Acinetobacter baumannii: clinical characteristics, epidemiology and pathogenesis. Expert Rev Anti Infect Ther 13:567–573. doi:10.1586/14787210.2015.1025055 25850806

[3] Xu A, Zhu H, Gao B, Weng H, Ding Z, Li M, Weng X, He G. 2020. Diagnosis of severe community-acquired pneumonia caused by Acinetobacter baumannii through next-generation sequencing: a case report. BMC Infect Dis 20:45. doi:10.1186/s12879-019-4733-5 31941459 PMC6964051

[4] Tacconelli E, Carrara E, Savoldi A, Harbarth S, Mendelson M, Monnet DL, Pulcini C, Kahlmeter G, Kluytmans J, Carmeli Y, Ouellette M, Outterson K, Patel J, Cavaleri M, Cox EM, Houchens CR, Grayson ML, Hansen P, Singh N, Theuretzbacher U, Magrini N, WHO Pathogens Priority List Working Group. 2018. Discovery, research, and development of new antibiotics: the WHO priority list of antibiotic-resistant bacteria and tuberculosis. Lancet Infect Dis 18:318–327. doi:10.1016/S1473-3099(17)30753-3 29276051

[5] Boral J, Genç Z, Pınarlık F, Ekinci G, Kuskucu MA, İrkören P, Kapmaz M, Tekin S, Çakar N, Şentürk E, Yurdakul F, Dikenelli B, Can F, Ergonul O. 2022. The association between Acinetobacter baumannii infections and the COVID-19 pandemic in an intensive care unit. Sci Rep 12:20808. doi:10.1038/s41598-022-25493-8 36460749 PMC9716169

[6] Hernández-González IL, Mateo-Estrada V, Castillo-Ramirez S. 2022. The promiscuous and highly mobile resistome of Acinetobacter baumannii. Microb Genom 8:000762. doi:10.1099/mgen.0.000762 35075990 PMC8914355

[7] Diancourt L, Passet V, Nemec A, Dijkshoorn L, Brisse S. 2010. The population structure of Acinetobacter baumannii: expanding multiresistant clones from an ancestral susceptible genetic pool. PLoS One 5:e10034. doi:10.1371/journal.pone.0010034 20383326 PMC2850921

[8] Kenyon JJ, Nigro SJ, Hall RM. 2014. Variation in the OC locus of Acinetobacter baumannii genomes predicts extensive structural diversity in the lipooligosaccharide. PLoS One 9:e107833. doi:10.1371/journal.pone.0107833 25247305 PMC4172580

[9] Wyres KL, Cahill SM, Holt KE, Hall RM, Kenyon JJ. 2020. Identification of Acinetobacter baumannii loci for capsular polysaccharide (KL) and lipooligosaccharide outer core (OCL) synthesis in genome assemblies using curated reference databases compatible with Kaptive. Microb Genom 6:e000339. doi:10.1099/mgen.0.000339 32118530 PMC7200062

[10] Repizo GD, Espariz M, Seravalle JL, Díaz Miloslavich JI, Steimbrüch BA, Shuman HA, Viale AM. 2020. Acinetobacter baumannii NCIMB8209: a rare environmental strain displaying extensive insertion sequence-mediated genome remodeling resulting in the loss of exposed cell structures and defensive mechanisms. mSphere 5:e00404-20. doi:10.1128/mSphere.00404-20 32727858 PMC7392541

[11] Repizo GD, Viale AM, Borges V, Cameranesi MM, Taib N, Espariz M, Brochier-Armanet C, Gomes JP, Salcedo SP. 2017. The environmental Acinetobacter baumannii isolate DSM30011 reveals clues into the preantibiotic era genome diversity, virulence potential, and niche range of a predominant nosocomial pathogen. Genome Biol Evol 9:2292–2307. doi:10.1093/gbe/evx162 28934377 PMC5604120

[12] Sykes EME, Mateo-Estrada V, Zhanel G, Dettman J, Chapados J, Gerdis S, Akineden Ö, Khan IIU, Castillo-Ramírez S, Kumar A. 2023. Emergence of ADC-5 cephalosporinase in environmental Acinetobacter baumannii from a German tank milk with a novel sequence type. Access Microbiol 5:acmi000485.v3. doi:10.1099/acmi.0.000485.v3 PMC10323797 37424542

[13] Mateo-Estrada V, Tyrrell C, Evans BA, Aguilar-Vera A, Drissner D, Castillo-Ramirez S, Walsh F. 2023. Acinetobacter baumannii from grass: novel but non-resistant clones. Microb Genom 9:mgen001054. doi:10.1099/mgen.0.001054 37439781 PMC10438806

[14] Hernández-González IL, Castillo-Ramírez S. 2020. Antibiotic-resistant Acinetobacter baumannii is a One Health problem. Lancet Microbe 1:e279. doi:10.1016/S2666-5247(20)30167-1 35544213

[15] Singh M, De Silva PM, Al-Saadi Y, Switala J, Loewen PC, Hausner G, Chen W, Hernandez I, Castillo-Ramirez S, Kumar A. 2020. Characterization of extremely drug-resistant and hypervirulent Acinetobacter baumannii AB030. Antibiotics (Basel) 9:328. doi:10.3390/antibiotics9060328 32560407 PMC7345994

[16] Fernando D, Zhanel G, Kumar A. 2013. Antibiotic resistance and expression of resistance-nodulation-division pump- and outer membrane porin-encoding genes in Acinetobacter species isolated from Canadian hospitals. Can J Infect Dis Med Microbiol 24:17–21. doi:10.1155/2013/696043 24421787 PMC3630023

[17] Teelucksingh T, Thompson LK, Cox G. 2020. The evolutionary conservation of Escherichia coli drug efflux pumps supports physiological functions. J Bacteriol 202:e00367-20. doi:10.1128/JB.00367-20 32839176 PMC7585057

[18] Rosenfeld N, Bouchier C, Courvalin P, Périchon B. 2012. Expression of the resistance-nodulation-cell division pump AdeIJK in Acinetobacter baumannii is regulated by AdeN, a TetR-type regulator. Antimicrob Agents Chemother 56:2504–2510. doi:10.1128/AAC.06422-11 22371895 PMC3346617

[19] Peleg AY, Jara S, Monga D, Eliopoulos GM, Moellering RC, Mylonakis E. 2009. Galleria mellonella as a model system to study Acinetobacter baumannii pathogenesis and therapeutics. Antimicrob Agents Chemother 53:2605–2609. doi:10.1128/AAC.01533-08 19332683 PMC2687231

[20] Cahill SM, Hall RM, Kenyon JJ. 2022. An update to the database for Acinetobacter baumannii capsular polysaccharide locus typing extends the extensive and diverse repertoire of genes found at and outside the K locus. Microb Genom 8:mgen000878. doi:10.1099/mgen.0.000878 36214673 PMC9676051

[21] Boyd DA, Mataseje LF, Pelude L, Mitchell R, Bryce E, Roscoe D, Embree J, Katz K, Kibsey P, Lavallee C, Simor AE, Taylor G, Turgeon N, Langley JM, Amaratunga K, Mulvey MR, Canadian Nosocomial Infection Surveillance P. 2019. Results from the Canadian nosocomial infection surveillance program for detection of carbapenemase-producing Acinetobacter spp. in Canadian hospitals, 2010-16. J Antimicrob Chemother 74:315–320. doi:10.1093/jac/dky416 30312401

[22] Gaiarsa S, Batisti Biffignandi G, Esposito EP, Castelli M, Jolley KA, Brisse S, Sassera D, Zarrilli R. 2019. Comparative analysis of the two Acinetobacter baumannii multilocus sequence typing (MLST) schemes. Front Microbiol 10:930. doi:10.3389/fmicb.2019.00930 31130931 PMC6510311

[23] Sorbello BM, Cahill SM, Kenyon JJ. 2023. Identification of further variation at the lipooligosaccharide outer core locus in Acinetobacter baumannii genomes and extension of the OCL reference sequence database for Kaptive. Microb Genom 9:mgen001042. doi:10.1099/mgen.0.001042 37310786 PMC10327509

[24] Munita JM, Arias CA. 2016. Mechanisms of antibiotic resistance. Microbiol Spectr 4. doi:10.1128/microbiolspec.VMBF-0016-2015 PMC4888801 27227291

[25] Evans BA, Amyes SGB. 2014. OXA β-lactamases. Clin Microbiol Rev 27:241–263. doi:10.1128/CMR.00117-13 24696435 PMC3993105

[26] Karah N, Dwibedi CK, Sjöström K, Edquist P, Johansson A, Wai SN, Uhlin BE. 2016. Novel aminoglycoside resistance transposons and transposon-derived circular forms detected in carbapenem-resistant Acinetobacter baumannii clinical isolates. Antimicrob Agents Chemother 60:1801–1818. doi:10.1128/AAC.02143-15 26824943 PMC4776018

[27] Girlich D, Poirel L, Nordmann P. 2010. First isolation of the bla_OXA-23_ carbapenemase gene from an environmental Acinetobacter baumannii isolate. Antimicrob Agents Chemother 54:578–579. doi:10.1128/AAC.00861-09 19884362 PMC2798507

[28] Hrenovic J, Durn G, Goic-Barisic I, Kovacic A. 2014. Occurrence of an environmental Acinetobacter baumannii strain similar to a clinical isolate in paleosol from Croatia. Appl Environ Microbiol 80:2860–2866. doi:10.1128/AEM.00312-14 24584245 PMC3993276

[29] Migliaccio A, Esposito EP, Bagattini M, Berisio R, Triassi M, De Gregorio E, Zarrilli R. 2021. Inhibition of AdeB, AceI, and AmvA efflux pumps restores chlorhexidine and benzalkonium susceptibility in Acinetobacter baumannii ATCC 19606. Front Microbiol 12:790263. doi:10.3389/fmicb.2021.790263 35197939 PMC8859242

[30] Coyne S, Rosenfeld N, Lambert T, Courvalin P, Périchon B. 2010. Overexpression of resistance-nodulation-cell division pump AdeFGH confers multidrug resistance in Acinetobacter baumannii. Antimicrob Agents Chemother 54:4389–4393. doi:10.1128/AAC.00155-10 20696879 PMC2944555

[31] Yoon EJ, Courvalin P, Grillot-Courvalin C. 2013. RND-type efflux pumps in multidrug-resistant clinical isolates of Acinetobacter baumannii: major role for AdeABC overexpression and AdeRS mutations. Antimicrob Agents Chemother 57:2989–2995. doi:10.1128/AAC.02556-12 23587960 PMC3697384

[32] Kornelsen V, Kumar A. 2021. Update on multidrug resistance efflux pumps in Acinetobacter spp. Antimicrob Agents Chemother 65:e0051421. doi:10.1128/AAC.00514-21 33903107 PMC8218648

[33] Darby EM, Bavro VN, Dunn S, McNally A, Blair JMA. 2023. RND pumps across the genus Acinetobacter: AdeIJK is the universal efflux pump. Microb Genom 9:mgen000964. doi:10.1099/mgen.0.000964 36995182 PMC10132057

[34] Jacobs AC, Thompson MG, Black CC, Kessler JL, Clark LP, McQueary CN, Gancz HY, Corey BW, Moon JK, Si Y, et al.. 2014. AB5075, a highly virulent isolate of Acinetobacter baumannii, as a model strain for the evaluation of pathogenesis and antimicrobial treatments. mBio 5:e01076-14. doi:10.1128/mBio.01076-14 24865555 PMC4045072

[35] Whiteway C, Valcek A, Philippe C, Strazisar M, De Pooter T, Mateus I, Breine A, Van der Henst C. 2022. Scarless excision of an insertion sequence restores capsule production and virulence in Acinetobacter baumannii. ISME J 16:1473–1477. doi:10.1038/s41396-021-01179-3 34949784 PMC9038732

[36] Repizo GD, Gagné S, Foucault-Grunenwald M-L, Borges V, Charpentier X, Limansky AS, Gomes JP, Viale AM, Salcedo SP. 2015. Differential role of the T6SS in Acinetobacter baumannii virulence. PLoS One 10:e0138265. doi:10.1371/journal.pone.0138265 26401654 PMC4581634

[37] Ronish LA, Lillehoj E, Fields JK, Sundberg EJ, Piepenbrink KH. 2019. The structure of PilA from Acinetobacter baumannii AB5075 suggests a mechanism for functional specialization in Acinetobacter type IV pili. J Biol Chem 294:218–230. doi:10.1074/jbc.RA118.005814 30413536 PMC6322890

[38] Harding CM, Hennon SW, Feldman MF. 2018. Uncovering the mechanisms of Acinetobacter baumannii virulence. Nat Rev Microbiol 16:91–102. doi:10.1038/nrmicro.2017.148 29249812 PMC6571207

[39] Lucidi M, Visaggio D, Migliaccio A, Capecchi G, Visca P, Imperi F, Zarrilli R. 2024. Pathogenicity and virulence of Acinetobacter baumannii: factors contributing to the fitness in healthcare settings and the infected host. Virulence 15:2289769. doi:10.1080/21505594.2023.2289769 38054753 PMC10732645

[40] Boone RL, Whitehead B, Avery TM, Lu J, Francis JD, Guevara MA, Moore RE, Chambers SA, Doster RS, Manning SD, Townsend SD, Dent L, Marshall D, Gaddy JA, Damo SM. 2021. Analysis of virulence phenotypes and antibiotic resistance in clinical strains of Acinetobacter baumannii isolated in Nashville, Tennessee. BMC Microbiol 21:21. doi:10.1186/s12866-020-02082-1 33422000 PMC7796680

[41] Tilley D, Law R, Warren S, Samis JA, Kumar A. 2014. CpaA a novel protease from Acinetobacter baumannii clinical isolates deregulates blood coagulation. FEMS Microbiol Lett 356:53–61. doi:10.1111/1574-6968.12496 24910020

[42] Waack U, Warnock M, Yee A, Huttinger Z, Smith S, Kumar A, Deroux A, Ginsburg D, Mobley HLT, Lawrence DA, Sandkvist M. 2018. CpaA is a glycan-specific adamalysin-like protease secreted by Acinetobacter baumannii that inactivates coagulation factor XII. mBio 9:e01606-18. doi:10.1128/mBio.01606-18 30563903 PMC6299215

[43] Haurat MF, Scott NE, Di Venanzio G, Lopez J, Pluvinage B, Boraston AB, Ferracane MJ, Feldman MF. 2020. The glycoprotease CpaA secreted by medically relevant Acinetobacter species targets multiple O-linked host glycoproteins. mBio 11:e02033-20. doi:10.1128/mBio.02033-20 33024038 PMC7542363

[44] Peterson E, Kaur P. 2018. Antibiotic resistance mechanisms in bacteria: relationships between resistance determinants of antibiotic producers, environmental bacteria, and clinical pathogens. Front Microbiol 9:2928. doi:10.3389/fmicb.2018.02928 30555448 PMC6283892

[45] Wright GD. 2007. The antibiotic resistome: the nexus of chemical and genetic diversity. Nat Rev Microbiol 5:175–186. doi:10.1038/nrmicro1614 17277795

[46] Cox G, Wright GD. 2013. Intrinsic antibiotic resistance: mechanisms, origins, challenges and solutions. Int J Med Microbiol 303:287–292. doi:10.1016/j.ijmm.2013.02.009 23499305

[47] Pritchard L, Glover RH, Humphris S, Elphinstone JG, Toth IK. 2016. Genomics and taxonomy in diagnostics for food security: soft-rotting enterobacterial plant pathogens. Anal Methods 8:12–24. doi:10.1039/C5AY02550H

[48] Kurtz S, Phillippy A, Delcher AL, Smoot M, Shumway M, Antonescu C, Salzberg SL. 2004. Versatile and open software for comparing large genomes. Genome Biol 5:R12. doi:10.1186/gb-2004-5-2-r12 14759262 PMC395750

[49] Graña-Miraglia L, Evans BA, López-Jácome LE, Hernández-Durán M, Colín-Castro CA, Volkow-Fernández P, Cevallos MA, Franco-Cendejas R, Castillo-Ramírez S. 2020. Origin of OXA-23 variant OXA-239 from a recently emerged lineage of Acinetobacter baumannii international clone V. mSphere 5:00801–00819. doi:10.1128/mSphere.00801-19 PMC6952199 31915222

[50] Page AJ, Cummins CA, Hunt M, Wong VK, Reuter S, Holden MTG, Fookes M, Falush D, Keane JA, Parkhill J. 2015. Roary: rapid large-scale prokaryote pan genome analysis. Bioinformatics 31:3691–3693. doi:10.1093/bioinformatics/btv421 26198102 PMC4817141

[51] Seemann T. Abricate, Github

[52] Zhanel GG, Adam HJ, Baxter MR, Fuller J, Nichol KA, Denisuik AJ, Golden AR, Hink R, Lagacé-Wiens PRS, Walkty A, Mulvey MR, Schweizer F, Bay D, Hoban DJ, Karlowsky JA, Alliance CAR C. 2019. 42936 pathogens from Canadian hospitals: 10 years of results (2007–16) from the CANWARD surveillance study. J Antimicrob Chemother 74:iv5–iv21. doi:10.1093/jac/dkz283 31505641

[53] Wayne P. 2019. Performance standards for antimicrobial susceptibility testing. In CLSI supplement M100, 29th ed. Clinical and laboratory standards Institute.

[54] Alcock BP, Raphenya AR, Lau TTY, Tsang KK, Bouchard M, Edalatmand A, Huynh W, Nguyen A-LV, Cheng AA, Liu S, et al.. 2020. CARD 2020: antibiotic resistome surveillance with the comprehensive antibiotic resistance database. Nucleic Acids Res 48:D517–D525. doi:10.1093/nar/gkz935 31665441 PMC7145624

[55] Gu Z, Eils R, Schlesner M. 2016. Complex heatmaps reveal patterns and correlations in multidimensional genomic data. Bioinformatics 32:2847–2849. doi:10.1093/bioinformatics/btw313 27207943

[56] Chen L, Zheng D, Liu B, Yang J, Jin Q. 2016. VFDB 2016: hierarchical and refined dataset for big data analysis—10 years on. Nucleic Acids Res 44:D694–D697. doi:10.1093/nar/gkv1239 26578559 PMC4702877

