# Supplementary material for: Phylogenomic and phenotypic analyses highlight the diversity of antibiotic resistance and virulence in both human and non-human Acinetobacter baumannii
Source: mSphere. 2024 Mar 5;9(3):e00741-23. doi: 10.1128/msphere.00741-23 (PMC10964423; doi:10.1128/msphere.00741-23)
Supplement: Supplemental Figures — Figures S1-S6. [file msphere.00741-23-s0001.pdf]

Supplement Figures

Comparative phylogenomic and phenotypic analysis highlights diversity of antibiotic resistance and virulence in *Acinetobacter baumannii*

Figure 1a – AdeN Dimerization Domain

```
  @
  @
logo               PEQTSLKDYLIQTLIRFYKHIIQPEHIAFLRLVIEQTQCNATLSQYLYEKCALDVQNTIAQALLISHQSGEITCTSPDHSSLMYFGILRDIEWRMIMGMPLPPNE
AB052-MST-SNC-8    PEQTSLKDYLIQTLIRFYKHIIQPEHIAFLRLVIEQTQCNATLSQYLYEKCALDVQNTIAQALLISHQSGEIICTSPDHSSLMYFGILRDIEWRMIMGMPLPPNE   196
AB428-AcS20        PEQTSLKDYLIQTLIRFYKHIIQPEHIAFLRLVIE......................................................................   126
AB422-MST-SNC-24   PEQTSLKDYLIQTLIRFYKHIIQPEHIAFLRLVIEQTQCNATLSQYLYEKCALDVQNTIAQALLISHQSGEITCTSPDHSSLMYFGILRDIEWRMIMGMPLPPNE   196
AB220-IK38         PEQTSLKDYLIQTLIRFYKHIIQPEHIAFLRLVIEQTQCNATLSQYLYEKCALDVQNTIAQALLISHQSGEITCTSPDHSSLMYFGILRDIEWRMIMGMPLPPNE   196
AB215-IK33         PEQTSLKDYLIQTLIRFYKHIIQPEHIAFLRLVIEQTQCNATLSQYLYEKCALDVQNTIAQALLISHQSGEITCTSPDHSSLMYFGILRDIEWRMIMGMPLPPHE   196
AB421-MST-SNC-9    PEQTSLKDYLIQTLIRFYKHIIQPEHIAFLRLVIEQTQCNATLSQYLYEKCALDVQNTIAQALLISHQSSEITCTSPDHSSLMYFGILRDIEWRMIMGMPLPPNE   196
AB046              PEQTSLKDYLIQTLIRFYKHIIQPEHIAFLRLVIEQTQCNATLSQYLYEKCALDVQNTIAQALLISHQSGEITCTSPDHSSLMYFGILRDIEWRMIMGMPLPPNE   196
AB338-IK12         PEQTSLKDYLIQTLIRFYKHIIQPEHIAFLRLVIEQTQCNATLSQYLYEKCALDVQNTIAQALLISHQSGEITCTSPDHSSLMYFGILRDIEWRMIMGMPLPPNE   196
AB341-IK15         PEQTSLKDYLIQTLIRFYKHIIQPEHIAFLRLVIEQTQCNATLSQYLYEKCALDVQNTIAQALLISHQSGEITCTSPDHSSLMYFGILRDIEWRMIMGMPLPPNE   196
AB345-IK19         PEQTSLKDYLIQTLIRFYKHIIQPEHIAFLRLVIEQTQCNATLSQYLYEKCALDVQNTIAQALLISHQSGEITCTSPDHSSLMYFGILRDIEWRMIMGMPLPPNE   196
AB351-IK26         PEQTSLKDYLIQTLIRFYKHIIQPEHIAFLRLVIEQTQCNATLSQYLYEKCALDVQNTIAQALLISHQSGEITCTSPDHSSLMYFGILRDIEWRMIMGMPLPPNE   196
AB354-IK29         PEQTSLKDYLIQTLIRFYKHIIQPEHIAFLRLVIEQTQCNATLSQYLYEKCALDVQNTIAQALLISHQSGEITCTSPDHSSLMYFGILRDIEWRMIMGMPLPPNE   196
AB224-IK42         PEQTSLKDYLIQTLIRFYKHIIQPEHIAFLRLVIEQTQCNATLSQYLYEKCALDVQNTIAQALLISHQSGEITCTSPDHSSLMYFGILRDIEWRMIMGMPLPPNE   196
AB424-AcS9         PEQTSLKDYLIQTLIRFYKHIIQPEHIAFLRLVIEQTQCNATLSQYLYEKCALDVQNTIAQALLISHQSGEITCTSPDHSSLMYFGILRDIEWRMIMGMPLPPNE   196
AB427-AcS19        PEQTSLKDYLIQTLIRFYKHIIQPEHIAFLRLVIEQTQCNATLSQYLYEKCALDVQNTIAQALLISHQSGEITCTSPDHSSLMYFGILRDIEWRMIMGMPLPPNE   196
ATCC17978          PEQTSLKDYLIQTLIRFYKHIIQPEHIAFLRLVIEQTQCNATLSQYLYEKCALDVQNTIAQALLISHQSGEITCTSPDHSSLMYFGILRDIEWRMIMGMPLPPNE   196
AB216-IK34         PEQTSLKDYLIQTLIRFYKHIIQPEHIAFLRLVIEQTQCNATLSQYLYEKCALDVQNTIAQALLISHQSGEITCTSPDHSSLMYFGILRDIEWRMIMGMPLPPNE   179
AB030              PEQTSLKDYLIQTLIRFYKHIIQPEHIAFLRLVIEQTQCNATLSQYLYEKCALDVQNTIAQALLISHQSGEITCTSPDHSSLMYFGILRDIEWRMIMGMPLPPNE   196
AB337-IK11         PEQTSLKDYLIQTLIRFYKHIIQPEHIAFLRLVIEQTQCNATLSQYLYEKCALDVQNTIAQALLISHQSGEITCTSPDHSSLMYFGILRDIEWRMIMGMPLPPNE   196
AB339-IK13         PEQTSLKDYLIQTLIRFYKHIIQPEHIAFLRLVIEQTQCNATLSQYLYEKCALDVQNTIAQALLISHQSGEITCTSPDHSSLMYFGILRDIEWRMIMGMPLPPNE   196
AB342-IK16         PEQTSLKDYLIQTLIRFYKHIIQPEHIAFLRLVIEQTQCNATLSQYLYEKCALDVQNTIAQALLISHQSGEITCTSPDHSSLMYFGILRDIEWRMIMGMPLPPNE   196
AB346-IK20         PEQTSLKDYLIQTLIRFYKHIIQPEHIAFLRLVIEQTQCNATLSQYLYEKCALDVQNTIAQALLISHQSGEITCTSPDHSSLMYFGILRDIEWRMIMGMPLPPNE   196
AB352-IK27         PEQTSLKDYLIQTLIRFYKHIIQPEHIAFLRLVIEQTQCNATLSQYLYEKCALDVQNTIAQALLISHQSGEITCTSPDHSSLMYFGILRDIEWRMIMGMPLPPNE   196
AB214-IK32         PEQTSLKDYLIQTLIRFYKHIIQPEHIAFLRLVIEQTQCNATLSQYLYEKCALDVQNTIAQALLISHQSGEITCTSPDHSSLMYFGILRDIEWRMIMGMPLPPNE   196
AB420-MST-SNC-5    PEQTSLKDYLIQTLIRFYKHIIQPEHIAFLRLVIEQTQCNATLSQYLYEKCALDVQNTIAQALLISHQSGEITCTSPDHSSLMYFGILRDIEWRMIMGMPLPPNE   196
AB425-AcS17        PEQTSLKDYLIQTLIRFYKHIIQPEHIAFLRLVIEQTQCNATLSQYLYEKCALDVQNTIAQALLISHQSGEITCTSPDHSSLMYFGILRDIEWRMIMGMPLPPNE   196
AB429-AcS27        PEQTSLKDYLIQTLIRFYKHIIQPEHIAFLRLVIEQTQCNATLSQYLYEKCALDVQNTIAQALLISHQSGEITCTSPDHSSLMYFGILRDIEWRMIMGMPLPPNE   196
AB426-AcS18        PEQTSLKDYLIQTLIRFYKHIIQPEHIAFLRLVIEQTQCNATLSQYLYEKCALDVQNTIAQALLISHQSGEITCTSPDHSSLMYFGILRDIEWRMIMGMPLPPNE   196
AB423-MST-SNC-253  PEQTSLKDYLIQTLIRFYKHIIQPEHIAFLRLVIEQTQCNATLSQYLYEKCALDVQNTIAQALLISHQSGEITCTSPDHSSLMYFGILRDIEWRMIMGMPLPPNE   196
AB218-IK36         PEQTSLKDYLIQTLIRFYKHIIQPEHIAFLRLVIEQTQCNATLSQYLYEKCALDVQNTIAQALLISHQSGEITCTSPDHSSLMYFGILRDIEWRMIMGMPLPPNE   196
AB353-IK28         PEQTSLKDYLIQTLIRFYKHIIQPEHIAFLRLVIEQTQCNATLSQYLYEKCALDVQNTIAQALLISHQSGEITCTSPDHSSLMYFGILRDIEWRMIMGMPLPPNE   196
AB347-IK21         PEQTSLKDYLIQTLIRFYKHIIQPEHIAFLRLVIEQTQCNATLSQYLYEKCALDVQNTIAQALLISHQSGEITCTSPDHSSLMYFGILRDIEWRMIMGMPLPPNE   196
AB343-IK17         PEQTSLKDYLIQTLIRFYKHIIQPEHIAFLRLVIEQTQCNATLSQYLYEKCALDVQNTIAQALLISHQSGEITCTSPDHSSLMYFGILRDIEWRMIMGMPLPPNE   196
AB340-IK14         PEQTSLKDYLIQTLIRFYKHIIQPEHIAFLRLVIEQTQCNATLSQYLYEKCALDVQNTIAQALLISHQSGEITCTSPDHSSLMYFGILRDIEWRMIMGMPLPPNE   196
AB219-IK37         PERTSLKDYLIHTLIRFYKHIIQPEHIAFLRLVIEQTQCNATLSQYLYEKCALDVQNTIAQALLISHQSGEITCTSPDHSSLMYFGILRDIEWRMIMGMPLPPNE   196
AB223-IK41         PERTSLKDYLIHTLIRFYKHIIQPEHIAFLRLVIEQTQCNATLSQYLYEKCALDVQNTIAQALLISHQSGEITCTSPDHSSLMYFGILRDIEWRMIMGMPLPPNE   196
consensus          !!*!!!!!!!!*!!!!!!!!!!!!!!!!!!!!!!!**********************************************************************
```

```
logo               TEVIDYINYCVDIFLKGH
AB052-MST-SNC-8    MEVIDYINYCVDIFLKGH   214
AB428-AcS20        ..................   126
AB422-MST-SNC-24   MEVIDYINYCVDIFLKGH   214
AB220-IK38         AEVIDYINYCVDIFLKGH   214
AB215-IK33         TEVIDYINYCVDIFLKGH   214
AB421-MST-SNC-9    TEVIDYINYCVDIFLKGH   214
AB046              TEVIDYINYCVDIFLKGH   214
AB338-IK12         TEVIDYINYCVDIFLKGH   214
AB341-IK15         TEVIDYINYCVDIFLKGH   214
AB345-IK19         TEVIDYINYCVDIFLKGH   214
AB351-IK26         TEVIDYINYCVDIFLKGH   214
AB354-IK29         TEVIDYINYCVDIFLKGH   214
AB224-IK42         TEVIDYINYCVDIFLKGH   214
AB424-AcS9         TEVIDYINYCVDIFLKGH   214
AB427-AcS19        TEVIDYINYCVDIFLKGH   214
ATCC17978          TEVIDYINYCVDIFLKGH   214
AB216-IK34         TEVIDYINYCVDIFLKGH   197
AB030              TEVIDYINYCVDIFLKGH   214
AB337-IK11         TEVIDYINYCVDIFLKGH   214
AB339-IK13         TEVIDYINYCVDIFLKGH   214
AB342-IK16         TEVIDYINYCVDIFLKGH   214
AB346-IK20         TEVIDYINYCVDIFLKGH   214
AB352-IK27         TEVIDYINYCVDIFLKGH   214
AB214-IK32         TEVIDYINYCVDIFLKGH   214
AB420-MST-SNC-5    TEVIDYINYCVDIFLKGH   214
AB425-AcS17        TEVIDYINYCVDIFLKGH   214
AB429-AcS27        TEVIDYINYCVDIFLKGH   214
AB426-AcS18        TEVIDYINYCVDIFLKGH   214
AB423-MST-SNC-253  TEVIDYINYCVDIFLKGH   214
AB218-IK36         TEVIDYINYCVDIFLKGH   214
AB353-IK28         TEVIDYINYCVDIFLKGH   214
AB347-IK21         TEVIDYINYCVDIFLKGH   214
AB343-IK17         TEVIDYINYCVDIFLKGH   214
AB340-IK14         TEVIDYINYCVDIFLKGH   214
AB219-IK37         TEVIDYINYCVDIFLKGH   214
AB223-IK41         TEVIDYINYCVDIFLKGH   214
consensus          ******************
```

X non-conserved
X ≥50% conserved

Figure 1b – AdeN AA1-105

```
                     @
                     @
AB052-MST-SNC-8      MHDPVLESHHLVCEKPQTRRGIERRLALLLSATELFLEKGYDAVSLDDIVNHAGGSKTSIYKYFGNKDGLFTAICDYRREMFFKDICIAFQPEQTSLKDYLIQTL   105
AB428-AcS20          MHDPVLESHHLVCEKPQTRRGIERRLALLLSATELFLEKGYDAVSLDDIVNHAGGSKTSIYKYFGNKDGLFTAICDYRREMFFKDICIAFQPEQTSLKDYLIQTL   105
AB422-MST-SNC-24     MHDPVLESHHLVCEKPQTRRGIERRLALLLSATELFLEKGYDAVSLDDIVNHAGGSKTSIYKYFGNKDGLFTAICDYRREMFFKDICIAFQPEQTSLKDYLIQTL   105
AB220-IK38           MHDPVLESHHLVCEKPQTRRGIERRLALLLSATELFLEKGYDAVSLDDIVNHAGGSKTSIYKYFGNKDGLFTAICDYRREMFFKDICIAFQPEQTSLKDYLIQTL   105
AB215-IK33           MHDPVLESHHLVCEKPQTRRGIERRLALLLSATELFLEKGYDAVSLDDIVNHAGGSKTSIYKYFGNKDGLFTAICDYRREMFFKDICIAFQPEQTSLKDYLIQTL   105
AB421-MST-SNC-9      MHDPVLESHHLVCEKPQTRRGIERRLALLLSATELFLEKGYDAVSLDDIVNHAGGSKTSIYKYFGNKDGLFTAICDYRREMFFKDICIAFQPEQTSLKDYLIQTL   105
AB046                MHDPVLESHHLVCEKPQTRRGIERRLALLLSATELFLEKGYDAVSLDDIVNHAGGSKTSIYKYFGNKDGLFTAICDYRREMFFKDICIAFQPEQTSLKDYLIQTL   105
AB338-IK12           MHDPVLESHHLVCEKPQTRRGIERRLALLLSATELFLEKGYDAVSLDDIVNHAGGSKTSIYKYFGNKDGLFTAICDYRREMFFKDICIAFQPEQTSLKDYLIQTL   105
AB341-IK15           MHDPVLESHHLVCEKPQTRRGIERRLALLLSATELFLEKGYDAVSLDDIVNHAGGSKTSIYKYFGNKDGLFTAICDYRREMFFKDICIAFQPEQTSLKDYLIQTL   105
AB345-IK19           MHDPVLESHHLVCEKPQTRRGIERRLALLLSATELFLEKGYDAVSLDDIVNHAGGSKTSIYKYFGNKDGLFTAICDYRREMFFKDICIAFQPEQTSLKDYLIQTL   105
AB351-IK26           MHDPVLESHHLVCEKPQTRRGIERRLALLLSATELFLEKGYDAVSLDDIVNHAGGSKTSIYKYFGNKDGLFTAICDYRREMFFKDICIAFQPEQTSLKDYLIQTL   105
AB354-IK29           MHDPVLESHHLVCEKPQTRRGIERRLALLLSATELFLEKGYDAVSLDDIVNHAGGSKTSIYKYFGNKDGLFTAICDYRREMFFKDICIAFQPEQTSLKDYLIQTL   105
AB224-IK42           MHDPVLESHHLVCEKPQTRRGIERRLALLLSATELFLEKGYDAVSLDDIVNHAGGSKTSIYKYFGNKDGLFTAICDYRREMFFKDICIAFQPEQTSLKDYLIQTL   105
AB424-AcS9           MHDPVLESHHLVCEKPQTRRGIERRLALLLSATELFLEKGYDAVSLDDIVNHAGGSKTSIYKYFGNKDGLFTAICDYRREMFFKDICIAFQPEQTSLKDYLIQTL   105
AB427-AcS19          MHDPVLESHHLVCEKPQTRRGIERRLALLLSATELFLEKGYDAVSLDDIVNHAGGSKTSIYKYFGNKDGLFTAICDYRREMFFKDICIAFQPEQTSLKDYLIQTL   105
ATCC17978            MHDPVLESHHLVCEKPQTRRGIERRLALLLSATELFLEKGYDAVSLDDIVNHAGGSKTSIYKYFGNKDGLFTAICDYRREMFFKDICIAFQPEQTSLKDYLIQTL   105
AB216-IK34           MHDPVLESHHLVCEKPQTRRGIG.................YDAVSLDDIVNHAGGSKTSIYKYFGNKDGLFTAICDYRREMFFKDICIAFQPEQTSLKDYLIQTL    88
AB030                MHDPVLESHHLVCEKPQTRRGIERRLALLLSATELFLEKGYDAVSLDDIVNHAGGSKTSIYKYFGNKDGLFTAICDYRREMFFKDICIAFQPEQTSLKDYLIQTL   105
AB337-IK11           MHDPVLESHHLVCEKPQTRRGIERRLALLLSATELFLEKGYDAVSLDDIVNHAGGSKTSIYKYFGNKDGLFTAICDYRREMFFKDICIAFQPEQTSLKDYLIQTL   105
AB339-IK13           MHDPVLESHHLVCEKPQTRRGIERRLALLLSATELFLEKGYDAVSLDDIVNHAGGSKTSIYKYFGNKDGLFTAICDYRREMFFKDICIAFQPEQTSLKDYLIQTL   105
AB342-IK16           MHDPVLESHHLVCEKPQTRRGIERRLALLLSATELFLEKGYDAVSLDDIVNHAGGSKTSIYKYFGNKDGLFTAICDYRREMFFKDICIAFQPEQTSLKDYLIQTL   105
AB346-IK20           MHDPVLESHHLVCEKPQTRRGIERRLALLLSATELFLEKGYDAVSLDDIVNHAGGSKTSIYKYFGNKDGLFTAICDYRREMFFKDICIAFQPEQTSLKDYLIQTL   105
AB352-IK27           MHDPVLESHHLVCEKPQTRRGIERRLALLLSATELFLEKGYDAVSLDDIVNHAGGSKTSIYKYFGNKDGLFTAICDYRREMFFKDICIAFQPEQTSLKDYLIQTL   105
AB214-IK32           MHDPVLESHHLVCEKPQTRRGIERRLALLLSATELFLEKGYDAVSLDDIVNHAGGSKTSIYKYFGNKDGLFTAICDYRREMFFKDICIAFQPEQTSLKDYLIQTL   105
AB420-MST-SNC-5      MHDPVLESHHLVCEKPQTRRGIERRLALLLSATELFLEKGYDAVSLDDIVNHAGGSKTSIYKYFGNKDGLFTAICDYRREMFFKDICIAFQPEQTSLKDYLIQTL   105
AB425-AcS17          MHDPVLESHHLVCEKPQTRRGIERRLALLLSATELFLEKGYDAVSLDDIVNHAGGSKTSIYKYFGNKDGLFTAICDYRREMFFKDICIAFQPEQTSLKDYLIQTL   105
AB429-AcS27          MHDPVLESHHLVCEKPQTRRGIERRLALLLSATELFLEKGYDAVSLDDIVNHAGGSKTSIYKYFGNKDGLFTAICDYRREMFFKDICIAFQPEQTSLKDYLIQTL   105
AB426-AcS18          MHDPVLESHHLVCEKPQTRRGIERRLALLLSATELFLEKGYDAVSLDDIVNHAGGSKTSIYKYFGNKDGLFTAICDYRREMFFKDICIAFQPEQTSLKDYLIQTL   105
AB423-MST-SNC-253    MHDPVLESHHLVCEKPQTRRGIERRLALLLSATELFLEKGYDAVSLDDIVNHAGGSKTSIYKYFGNKDGLFTAICDYRREMFFKDICIAFQPEQTSLKDYLIQTL   105
AB218-IK36           MHDPVLESHHLVCEKPQTRRGIERRLALLLSATELFLEKGYDAVSLDDIVNHAGGSKTSIYKYFGNKDGLFTAICDYRREMFFKDICIAFQPEQTSLKDYLIQTL   105
AB353-IK28           MHDPVLESHHLVCEKPQTRRGIERRLALLLSATELFLEKGYDAVSLDDIVNHAGGSKTSIYKYFGNKDGLFTAICDYRREMFFKDICIAFQPEQTSLKDYLIQTL   105
AB347-IK21           MHDPVLESHHLVCEKPQTRRGIERRLALLLSATELFLEKGYDAVSLDDIVNHAGGSKTSIYKYFGNKDGLFTAICDYRREMFFKDICIAFQPEQTSLKDYLIQTL   105
AB343-IK17           MHDPVLESHHLVCEKPQTRRGIERRLALLLSATELFLEKGYDAVSLDDIVNHAGGSKTSIYKYFGNKDGLFTAICDYRREMFFKDICIAFQPEQTSLKDYLIQTL   105
AB340-IK14           MHDPVLESHHLVCEKPQTRRGIERRLALLLSATELFLEKGYDAVSLDDIVNHAGGSKTSIYKYFGNKDGLFTAICDYRREMFFKDICIAFQPEQTSLKDYLIQTL   105
AB219-IK37           MHDPVLESHHLVCEKLQTRRGIERRLALLLSATELFLEKGYDAVSLDDIVNHAGGSKTSIYKYFGNKDGLFTAICDYRREMFFKDICIAFQPERTSLKDYLIHTL   105
AB223-IK41           MHDPVLESHHLVCEKLQTRRGIERRLALLLSATELFLEKGYDAVSLDDIVNHAGGSKTSIYKYFGNKDGLFTAICDYRREMFFKDICIAFQPERTSLKDYLIHTL   105
consensus            !!!!!!!!!!!!!!!*!!!!!!******************!!!!!!!!!!!!!!!!!!!!!!!!!!!!!!!!!!!!!!!!!!!!!!!!!!!!!*!!!!!!!!*!!
```

Figure 2a – AdeR DNA binding domain

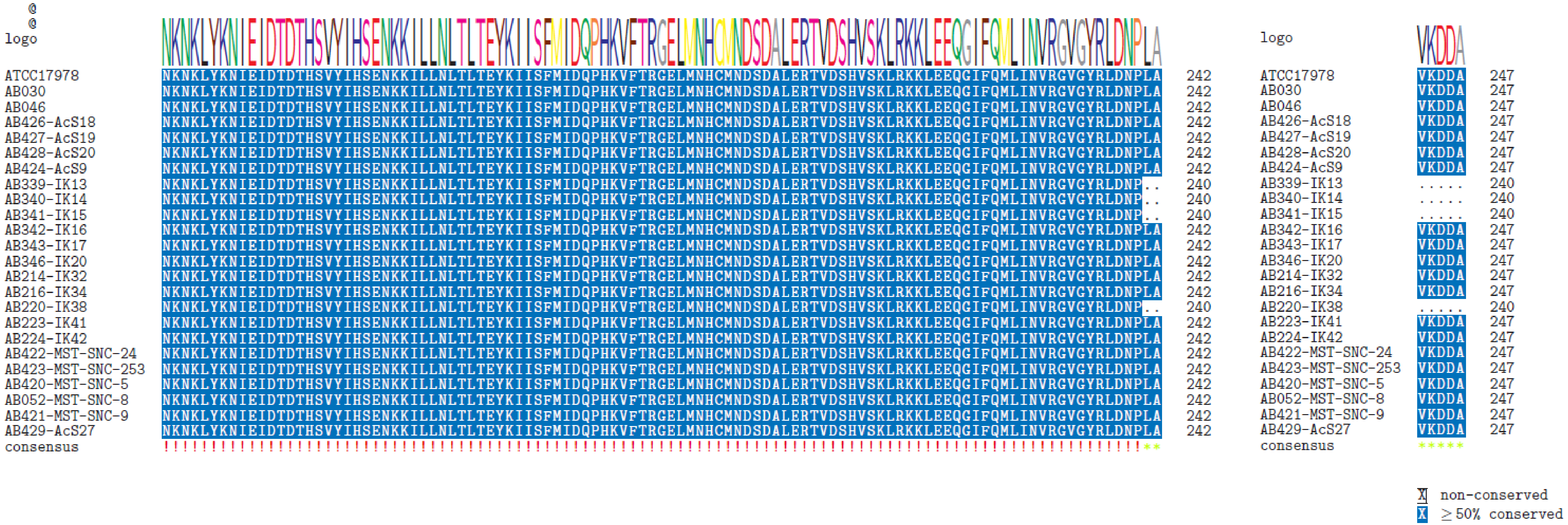

Figure 2b – AdeR of AB220-IK38, AB341-IK15, AB340-IK14 and AB339-IK13

```
   @
   @
AB339-IK13  MFDHSFSFDCQDKVILVVEDDYDIGDIIENYLKREGMSVIRAMNGKQAIELHASQPIDLILLDIKLPELNGWEVLNKIRQKAQTPVIMLTALDQDIDKVMALRIGADDFV  110
AB340-IK14  MFDHSFSFDCQDKVILVVEDDYDIGDIIENYLKREGMSVIRAMNGKQAIELHASQPIDLILLDIKLPELNGWEVLNKIRQKAQTPVIMLTALDQDIDKVMALRIGADDFV  110
AB220-IK38  MFDHSFSFDCQDKVILVVEDDYDIGDIIENYLKREGMSVIRAMNGKQAIELHASQPIDLILLDIKLPELNGWEVLNKIRQKAQTPVIMLTALDQDIDKVMALRIGADDFV  110
AB341-IK15  MFDHSFSFDCQDKVILVVEDDYDIGDIIENYLKREGMSVIRAMNGKQAIELHASQPIDLILLDIKLPELNGWEVLNKIRQKAQTPVIMLTALDQDIDKVMALRIGADDFV  110
consensus   !!!!!!!!!!!!!!!!!!!!!!!!!!!!!!!!!!!!!!!!!!!!!!!!!!!!!!!!!!!!!!!!!!!!!!!!!!!!!!!!!!!!!!!!!!!!!!!!!!!!!!!!!!!!!!

AB339-IK13  VKPFNPNEVVARVQAVLRRTQFANKATNKNKLYKNIEIDTDTHSVYIHSENKKILLNLTLTEYKIISFMIDQPHKVFTRGELMNHCMNDSDALERTVDSHVSKLRKKLEE  220
AB340-IK14  VKPFNPNEVVARVQAVLRRTQFANKATNKNKLYKNIEIDTDTHSVYIHSENKKILLNLTLTEYKIISFMIDQPHKVFTRGELMNHCMNDSDALERTVDSHVSKLRKKLEE  220
AB220-IK38  VKPFNPNEVVARVQAVLRRTQFANKATNKNKLYKNIEIDTDTHSVYIHSENKKILLNLTLTEYKIISFMIDQPHKVFTRGELMNHCMNDSDALERTVDSHVSKLRKKLEE  220
AB341-IK15  VKPFNPNEVVARVQAVLRRTQFANKATNKNKLYKNIEIDTDTHSVYIHSENKKILLNLTLTEYKIISFMIDQPHKVFTRGELMNHCMNDSDALERTVDSHVSKLRKKLEE  220
consensus   !!!!!!!!!!!!!!!!!!!!!!!!!!!!!!!!!!!!!!!!!!!!!!!!!!!!!!!!!!!!!!!!!!!!!!!!!!!!!!!!!!!!!!!!!!!!!!!!!!!!!!!!!!!!!!

AB339-IK13  QGIFQMLINVRGVGYRLDNP  240
AB340-IK14  QGIFQMLINVRGVGYRLDNP  240
AB220-IK38  QGIFQMLINVRGVGYRLDNP  240
AB341-IK15  QGIFQMLINVRGVGYRLDNP  240
consensus   !!!!!!!!!!!!!!!!!!!!

            X  non-conserved
            X  ≥50% conserved
```

Figure 2c – BaeR

```
     @
     @
AB219-IK37          MKHIMLVEDEVELAHLVRDYLEAAGFEVSMFHDGQDAYTSFQQRKPNLMILDLMVPRMDGLTICHKVREQSDLPIIMVTARTEEIDRVLGLNMGADDYVCKPFSP   105
AB223-IK41          MKHIMLVEDEVELAHLVRDYLEAAGFEVSMFHDGQDAYTSFQQRKPNLMILDLMVPRMDGLTICHKVREQSDLPIIMVTARTEEIDRVLGLNMGADDYVCKPFSP   105
AB426-AcS18         MKHIMLVEDEVELAHLVRDYLEAAGFEVSMFHDGQDAYTSFQQRKPNLMILDLMVPRMDGLTICHKVREQSDLPIIMVTARTEEIDRVLGLNMGADDYVCKPFSP   105
AB428-AcS20         MKHIMLVEDEVELAHLVRDYLEAAGFEVSMFHDGQDAYTSFQQRKPNLMILDLMVPRMDGLTICHKVREQSDLPIIMVTARTEEIDRVLGLNMGADDYVCKPFSP   105
AB214-IK32          MKHIMLVEDEVELAHLVRDYLEAAGFEVSMFHDGQDAYTNFQQRKPNLMILDLMVPRMDGLTICRKVREQSDLPIIMVTARTEEIDRVLGLNMGADDYVCKPFSP   105
ATCC17978           MKHIMLVEDEVELAHLVRDYLEAAGFEVSMFHDGQDAYTSFQQRKPNLMILDLMVPRMDGLTICRKVREQSDLPIIMVTARTEEIDRVLGLNMGADDYVCKPFSP   105
AB030               MKHIMLVEDEVELAHLVRDYLEAAGFEVSMFHDGQDAYTSFQQRKPNLMILDLMVPRMDGLTICRKVREQSDLPIIMVTARTEEIDRVLGLNMGADDYVCKPFSP   105
AB046               MKHIMLVEDEVELAHLVRDYLEAAGFEVSMFHDGQDAYTSFQQRKPNLMILDLMVPRMDGLTICRKVREQSDLPIIMVTARTEEIDRVLGLNMGADDYVCKPFSP   105
AB337-IK11          MKHIMLVEDEVELAHLVRDYLEAAGFEVSMFHDGQDAYTSFQQRKPNLMILDLMVPRMDGLTICRKVREQSDLPIIMVTARTEEIDRVLGLNMGADDYVCKPFSP   105
AB338-IK12          MKHIMLVEDEVELAHLVRDYLEAAGFEVSMFHDGQDAYTSFQQRKPNLMILDLMVPRMDGLTICRKVREQSDLPIIMVTARTEEIDRVLGLNMGADDYVCKPFSP   105
AB339-IK13          MKHIMLVEDEVELAHLVRDYLEAAGFEVSMFHDGQDAYTSFQQRKPNLMILDLMVPRMDGLTICRKVREQSDLPIIMVTARTEEIDRVLGLNMGADDYVCKPFSP   105
AB340-IK14          MKHIMLVEDEVELAHLVRDYLEAAGFEVSMFHDGQDAYTSFQQRKPNLMILDLMVPRMDGLTICRKVREQSDLPIIMVTARTEEIDRVLGLNMGADDYVCKPFSP   105
AB341-IK15          MKHIMLVEDEVELAHLVRDYLEAAGFEVSMFHDGQDAYTSFQQRKPNLMILDLMVPRMDGLTICRKVREQSDLPIIMVTARTEEIDRVLGLNMGADDYVCKPFSP   105
AB342-IK16          MKHIMLVEDEVELAHLVRDYLEAAGFEVSMFHDGQDAYTSFQQRKPNLMILDLMVPRMDGLTICRKVREQSDLPIIMVTARTEEIDRVLGLNMGADDYVCKPFSP   105
AB343-IK17          MKHIMLVEDEVELAHLVRDYLEAAGFEVSMFHDGQDAYTSFQQRKPNLMILDLMVPRMDGLTICRKVREQSDLPIIMVTARTEEIDRVLGLNMGADDYVCKPFSP   105
AB345-IK19          MKHIMLVEDEVELAHLVRDYLEAAGFEVSMFHDGQDAYTSFQQRKPNLMILDLMVPRMDGLTICRKVREQSDLPIIMVTARTEEIDRVLGLNMGADDYVCKPFSP   105
AB346-IK20          MKHIMLVEDEVELAHLVRDYLEAAGFEVSMFHDGQDAYTSFQQRKPNLMILDLMVPRMDGLTICRKVREQSDLPIIMVTARTEEIDRVLGLNMGADDYVCKPFSP   105
AB347-IK21          MKHIMLVEDEVELAHLVRDYLEAAGFEVSMFHDGQDAYTSFQQRKPNLMILDLMVPRMDGLTICRKVREQSDLPIIMVTARTEEIDRVLGLNMGADDYVCKPFSP   105
AB351-IK26          MKHIMLVEDEVELAHLVRDYLEAAGFEVSMFHDGQDAYTSFQQRKPNLMILDLMVPRMDGLTICRKVREQSDLPIIMVTARTEEIDRVLGLNMGADDYVCKPFSP   105
AB352-IK27          MKHIMLVEDEVELAHLVRDYLEAAGFEVSMFHDGQDAYTSFQQRKPNLMILDLMVPRMDGLTICRKVREQSDLPIIMVTARTEEIDRVLGLNMGADDYVCKPFSP   105
AB215-IK33          MKHIMLVEDEVELAHLVRDYLEAAGFEVSMFHDGQDAYTSFQQRKPNLMILDLMVPRMDGLTICRKVREQSDLPIIMVTARTEEIDRVLGLNMGADDYVCKPFSP   105
AB216-IK34          MKHIMLVEDEVELAHLVRDYLEAAGFEVSMFHDGQDAYTSFQQRKPNLMILDLMVPRMDGLTICRKVREQSDLPIIMVTARTEEIDRVLGLNMGADDYVCKPFSP   105
AB218-IK36          MKHIMLVEDEVELAHLVRDYLEAAGFEVSMFHDGQDAYTSFQQRKPNLMILDLMVPRMDGLTICRKVREQSDLPIIMVTARTEEIDRVLGLNMGADDYVCKPFSP   105
AB220-IK38          MKHIMLVEDEVELAHLVRDYLEAAGFEVSMFHDGQDAYTSFQQRKPNLMILDLMVPRMDGLTICRKVREQSDLPIIMVTARTEEIDRVLGLNMGADDYVCKPFSP   105
AB224-IK42          MKHIMLVEDEVELAHLVRDYLEAAGFEVSMFHDGQDAYTSFQQRKPNLMILDLMVPRMDGLTICRKVREQSDLPIIMVTARTEEIDRVLGLNMGADDYVCKPFSP   105
AB420-MST-SNC-5     MKHIMLVEDEVELAHLVRDYLEAAGFEVSMFHDGQDAYTSFQQRKPNLMILDLMVPRMDGLTICRKVREQSDLPIIMVTARTEEIDRVLGLNMGADDYVCKPFSP   105
AB052-MST-SNC-8     MKHIMLVEDEVELAHLVRDYLEAAGFEVSMFHDGQDAYTSFQQRKPNLMILDLMVPRMDGLTICRKVREQSDLPIIMVTARTEEIDRVLGLNMGADDYVCKPFSP   105
AB421-MST-SNC-9     MKHIMLVEDEVELAHLVRDYLEAAGFEVSMFHDGQDAYTSFQQRKPNLMILDLMVPRMDGLTICRKVREQSDLPIIMVTARTEEIDRVLGLNMGADDYVCKPFSP   105
AB422-MST-SNC-24    MKHIMLVEDEVELAHLVRDYLEAAGFEVSMFHDGQDAYTSFQQRKPNLMILDLMVPRMDGLTICRKVREQSDLPIIMVTARTEEIDRVLGLNMGADDYVCKPFSP   105
AB423-MST-SNC-253   MKHIMLVEDEVELAHLVRDYLEAAGFEVSMFHDGQDAYTSFQQRKPNLMILDLMVPRMDGLTICRKVREQSDLPIIMVTARTEEIDRVLGLNMGADDYVCKPFSP   105
AB424-AcS9          MKHIMLVEDEVELAHLVRDYLEAAGFEVSMFHDGQDAYTSFQQRKPNLMILDLMVPRMDGLTICRKVREQSDLPIIMVTARTEEIDRVLGLNMGADDYVCKPFSP   105
AB425-AcS17         MKHIMLVEDEVELAHLVRDYLEAAGFEVSMFHDGQDAYTSFQQRKPNLMILDLMVPRMDGLTICRKVREQSDLPIIMVTARTEEIDRVLGLNMGADDYVCKPFSP   105
AB427-AcS19         MKHIMLVEDEVELAHLVRDYLEAAGFEVSMFHDGQDAYTSFQQRKPNLMILDLMVPRMDGLTICRKVREQSDLPIIMVTARTEEIDRVLGLNMGADDYVCKPFSP   105
AB429-AcS27         MKHIMLVEDEVELAHLVRDYLEAAGFEVSMFHDGQDAYTSFQQRKPNLMILDLMVPRMDGLTICRKVREQSDLPIIMVTARTEEIDRVLGLNMGADDYVCKPFSP   105
AB353-IK28          MKHIMLVEDEVELAHLVRDYLEAAGFEVSMFHDGQDAYTSFQQRKPNLMILDLMVPRMDGLTICRKVREQSDLPIIMVTARTEEIDRVLGLNMGADDYVCKPFSP   105
AB354-IK29          MKHIMLVEDEVELAHLVRDYLEAAGFEVSMFHDGQDAYTSFQQRKPNLMILDLMVPRMDGLTICRKVREQSDLPIIMVTARTEEIDRVLGLNMGADDYVCKPFSP   105
consensus           !!!!!!!!!!!!!!!!!!!!!!!!!!!!!!!!!!!!!!!*!!!!!!!!!!!!!!!!!!!!!!!!*!!!!!!!!!!!!!!!!!!!!!!!!!!!!!!!!!!!!!!!!
```

Figure 2c – BaeR

```
AB219-IK37          KELVARVQAVLRRLERKAEPEQNDSFRIDKAQQRIWYQQKSLSLTPTEFRLLELFLEHVGQVYSRAQLLDHINPDSFDVADRVIDSHIKNLRRKITEVAETGNRH  210
AB223-IK41          KELVARVQAVLRRLERKAEPEQNDSFRIDKAQQRIWYQQKSLSLTPTEFRLLELFLEHVGQVYSRAQLLDHINPDSFDVADRVIDSHIKNLRRKITEVAETGNRH  210
AB426-AcS18         KELVARVQAVLRRLERKAEPEQNDSFRIDKAQQRIWYQQKSLSLTPTEFRLLELFLEHVGQVYSRAQLLDHINPDSFDVADRVIDSHIKNLRRKITEVAETGNRH  210
AB428-AcS20         KELVARVQAVLRRLERKAEPEQNDSFRIDKAQQRIWYQQKSLSLTPTEFRLLELFLEHVGQVYSRAQLLDHINPDSFDVADRVIDSHIKNLRRKITEVAETGNRH  210
AB214-IK32          KELVARVQAVLRRLERKAEPEQNDSFRIDKAQQRIWYQQKSLSLTPTEFRLLELFLEHVGQVYSRAQLLDHINPDSFDVADRVIDSHIKNLRRKITEVAETGNRH  210
ATCC17978           KELVARVQAVLRRLERKAEPEQNDSFRIDKAQQRIWYQQKSLSLTPTEFRLLELFLEHVGQVYSRAQLLDHINPDSFDVADRVIDSHIKNLRRKITEVAETGNRH  210
AB030               KELVARVQAVLRRLERKAEPEQNDSFRIDKAQQRIWYQQKSLSLTPTEFRLLELFLEHVGQVYSRAQLLDHINPDSFDVADRVIDSHIKNLRRKITEVAETGNRH  210
AB046               KELVARVQAVLRRLERKAEPEQNDSFRIDKAQQRIWYQQKSLSLTPTEFRLLELFLEHVGQVYSRAQLLDHINPDSFDVADRVIDSHIKNLRRKITEVAETGNRH  210
AB337-IK11          KELVARVQAVLRRLERKAEPEQNDSFRIDKAQQRIWYQQKSLSLTPTEFRLLELFLEHVGQVYSRAQLLDHINPDSFDVADRVIDSHIKNLRRKITEVAETGNRH  210
AB338-IK12          KELVARVQAVLRRLERKAEPEQNDSFRIDKAQQRIWYQQKSLSLTPTEFRLLELFLEHVGQVYSRAQLLDHINPDSFDVADRVIDSHIKNLRRKITEVAETGNRH  210
AB339-IK13          KELVARVQAVLRRLERKAEPEQNDSFRIDKAQQRIWYQQKSLSLTPTEFRLLELFLEHVGQVYSRAQLLDHINPDSFDVADRVIDSHIKNLRRKITEVAETGNRH  210
AB340-IK14          KELVARVQAVLRRLERKAEPEQNDSFRIDKAQQRIWYQQKSLSLTPTEFRLLELFLEHVGQVYSRAQLLDHINPDSFDVADRVIDSHIKNLRRKITEVAETGNRH  210
AB341-IK15          KELVARVQAVLRRLERKAEPEQNDSFRIDKAQQRIWYQQKSLSLTPTEFRLLELFLEHVGQVYSRAQLLDHINPDSFDVADRVIDSHIKNLRRKITEVAETGNRH  210
AB342-IK16          KELVARVQAVLRRLERKAEPEQNDSFRIDKAQQRIWYQQKSLSLTPTEFRLLELFLEHVGQVYSRAQLLDHINPDSFDVADRVIDSHIKNLRRKITEVAETGNRH  210
AB343-IK17          KELVARVQAVLRRLERKAEPEQNDSFRIDKAQQRIWYQQKSLSLTPTEFRLLELFLEHVGQVYSRAQLLDHINPDSFDVADRVIDSHIKNLRRKITEVAETGNRH  210
AB345-IK19          KELVARVQAVLRRLERKAEPEQNDSFRIDKAQQRIWYQQKSLSLTPTEFRLLELFLEHVGQVYSRAQLLDHINPDSFDVADRVIDSHIKNLRRKITEVAETGNRH  210
AB346-IK20          KELVARVQAVLRRLERKAEPEQNDSFRIDKAQQRIWYQQKSLSLTPTEFRLLELFLEHVGQVYSRAQLLDHINPDSFDVADRVIDSHIKNLRRKITEVAETGNRH  210
AB347-IK21          KELVARVQAVLRRLERKAEPEQNDSFRIDKAQQRIWYQQKSLSLTPTEFRLLELFLEHVGQVYSRAQLLDHINPDSFDVADRVIDSHIKNLRRKITEVAETGNRH  210
AB351-IK26          KELVARVQAVLRRLERKAEPEQNDSFRIDKAQQRIWYQQKSLSLTPTEFRLLELFLEHVGQVYSRAQLLDHINPDSFDVADRVIDSHIKNLRRKITEVAETGNRH  210
AB352-IK27          KELVARVQAVLRRLERKAEPEQNDSFRIDKAQQRIWYQQKSLSLTPTEFRLLELFLEHVGQVYSRAQLLDHINPDSFDVADRVIDSHIKNLRRKITEVAETGNRH  210
AB215-IK33          KELVARVQAVLRRLERKAEPEQNDSFRIDKAQQRIWYQQKSLSLTPTEFRLLELFLEHVGQVYSRAQLLDHINPDSFDVADRVIDSHIKNLRRKITEVAETGNRH  210
AB216-IK34          KELVARVQAVLRRLERKAEPEQNDSFRIDKAQQRIWYQQKSLSLTPTEFRLLELFLEHVGQVYSRAQLLDHINPDSFDVADRVIDSHIKNLRRKITEVAETGNRH  210
AB218-IK36          KELVARVQAVLRRLERKAEPEQNDSFRIDKAQQRIWYQQKSLSLTPTEFRLLELFLEHVGQVYSRAQLLDHINPDSFDVADRVIDSHIKNLRRKITEVAETGNRH  210
AB220-IK38          KELVARVQAVLRRLERKAEPEQNDSFRIDKAQQRIWYQQKSLSLTPTEFRLLELFLEHVGQVYSRAQLLDHINPDSFDVADRVIDSHIKNLRRKITEVAETGNRH  210
AB224-IK42          KELVARVQAVLRRLERKAEPEQNDSFRIDKAQQRIWYQQKSLSLTPTEFRLLELFLEHVGQVYSRAQLLDHINPDSFDVADRVIDSHIKNLRRKITEVAETGNRH  210
AB420-MST-SNC-5     KELVARVQAVLRRLERKAEPEQNDSFRIDKAQQRIWYQQKSLSLTPTEFRLLELFLEHVGQVYSRAQLLDHINPDSFDVADRVIDSHIKNLRRKITEVAETGNRH  210
AB052-MST-SNC-8     KELVARVQAVLRRLERKAEPEQNDSFRIDKAQQRIWYQQKSLSLTPTEFRLLELFLEHVGQVYSRAQLLDHINPDSFDVADRVIDSHIKNLRRKITEVAETGNRH  210
AB421-MST-SNC-9     KELVARVQAVLRRLERKAEPEQNDSFRIDKAQQRIWYQQKSLSLTPTEFRLLELFLEHVGQVYSRAQLLDHINPDSFDVADRVIDSHIKNLRRKITEVAETGNRH  210
AB422-MST-SNC-24    KELVARVQAVLRRLERKAEPEQNDSFRIDKAQQRIWYQQKSLSLTPTEFRLLELFLEHVGQVYSRAQLLDHINPDSFDVADRVIDSHIKNLRRKITEVAETGNRH  210
AB423-MST-SNC-253   KELVARVQAVLRRLERKAEPEQNDSFRIDKAQQRIWYQQKSLSLTPTEFRLLELFLEHVGQVYSRAQLLDHINPDSFDVADRVIDSHIKNLRRKITEVAETGNRH  210
AB424-AcS9          KELVARVQAVLRRLERKAEPEQNDSFRIDKAQQRIWYQQKSLSLTPTEFRLLELFLEHVGQVYSRAQLLDHINPDSFDVADRVIDSHIKNLRRKITEVAETGNRH  210
AB425-AcS17         KELVARVQAVLRRLERKAEPEQNDSFRIDKAQQRIWYQQKSLSLTPTEFRLLELFLEHVGQVYSRAQLLDHINPDSFDVADRVIDSHIKNLRRKITEVAETGNRH  210
AB427-AcS19         KELVARVQAVLRRLERKAEPEQNDSFRIDKAQQRIWYQQKSLSLTPTEFRLLELFLEHVGQVYSRAQLLDHINPDSFDVADRVIDSHIKNLRRKITEVAETGNRH  210
AB429-AcS27         KELVARVQAVLRRLERKAEPEQNDSFRIDKAQQRIWYQQKSLSLTPTEFRLLELFLEHVGQVYSRAQLLDHINPDSFDVADRVIDSHIKNLRRKITEVAETGNRH  210
AB353-IK28          KELVARVQAVLRRLERKAEPEQNDSFRIDKAQQRIWYQQKSLSLTPTEFRLLELFLEHVGQVYSRAQLLDHINPDSFDVADRVIDSHIKNLRRKITEVAETGNRH  210
AB354-IK29          KELVARVQAVLRRLERKAEPEQNDSFRIDKAQQRIWYQQKSLSLTPTEFRLLELFLEHVGQVYSRAQLLDHINPDSFDVADRVIDSHIKNLRRKITEVAETGNRH  210
consensus           !!!!!!!!!!!!!!!!!!!!!!!!!!!!!!!!!!!!!!!!!!!!!!!!!!!!!!!!!!!!!!!!!!!!!!!!!!!!!!!!!!!!!!!!!!!!!!!!!!!!!!!!!
```

Figure 2c – BaeR

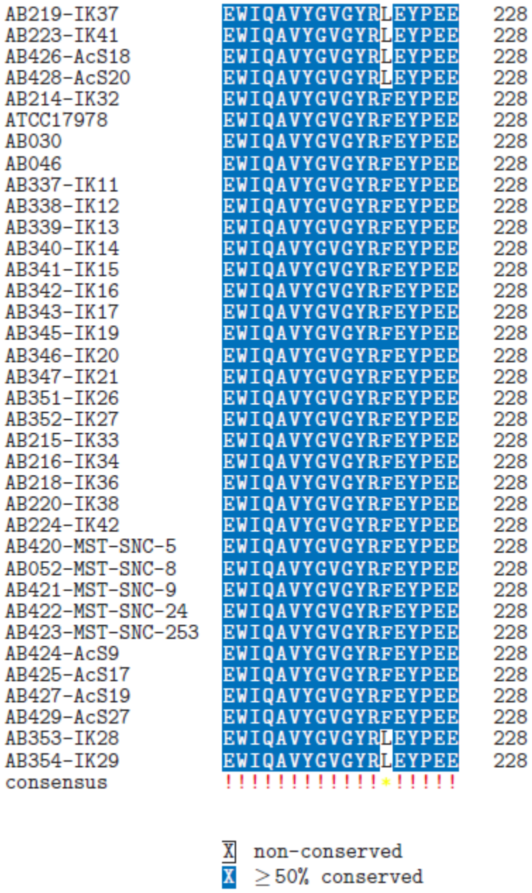

Figure 3 – AdeL

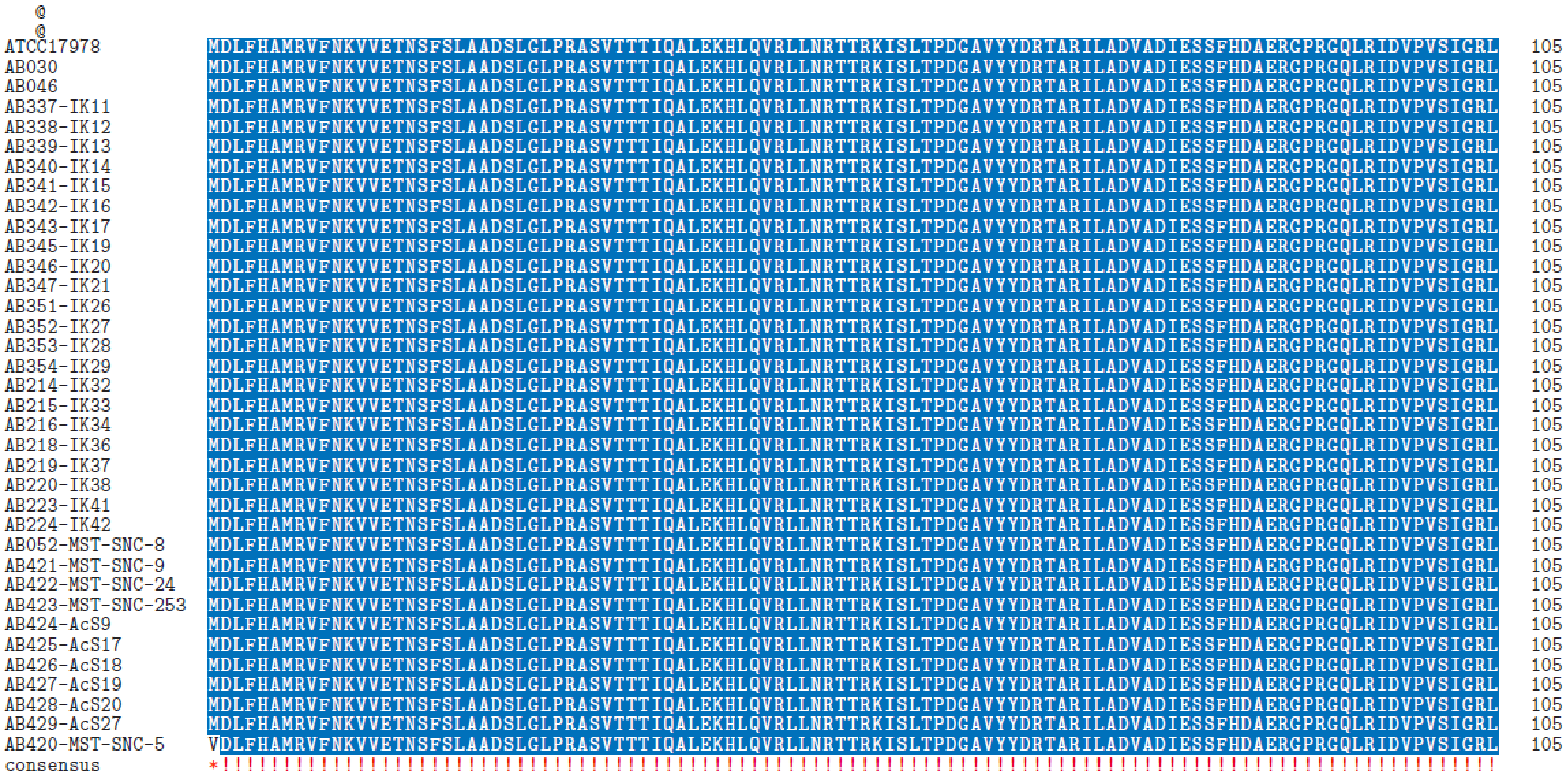

Figure 3 – AdeL

ATCC17978	ILIPRLRDFHARYPDIDLVIGLNDRPVDLVGEAVDCAIRVGELKDSSLIARRIGTFQCATAASPIYLEKYGEPTSIEDLQKNHKAIHFFSSRTGRNFDWDFVVDD	210
AB030	ILIPRLRDFHARYPDIDLVIGLNDRPVDLVGEAVDCAIRVGELKDSSLIARRIGTFQCATAASPIYLEKYGEPTSIEDLQKNHKAIHFFSS..............	196
AB046	ILIPRLRDFHARYPDIDLVIGLNDRPVDLVGEAVDCAIRVGELKDSSLIARRIGTFQCATAASPIYLEKYGEPTSIEDLQKNHKAIHFFSSRTGRNFDWDFVVDD	210
AB337-IK11	ILIPRLRDFHARYPDIDLVIGLNDRPVDLVGEAVDCAIRVGELKDSSLIARRIGTFQCATAASPIYLEKYGEPTSIEDLQKNHKAIHFFSSRTGRNFDWDFVVDD	210
AB338-IK12	ILIPRLRDFHARYPDIDLVIGLNDRPVDLVGEAVDCAIRVGELKDSSLIARRIGTFQCATAASPIYLEKYGEPTSIEDLQKNHKAIHFFSSRTGRNFDWDFVVDD	210
AB339-IK13	ILIPRLRDFHARYPDIDLVIGLNDRPVDLVGEAVDCAIRVGELKDSSLIARRIGTFQCATAASPIYLEKYGEPTSIEDLQKNHKAIHFFSSRTGRNFDWDFVVDD	210
AB340-IK14	ILIPRLRDFHARYPDIDLVIGLNDRPVDLVGEAVDCAIRVGELKDSSLIARRIGTFQCATAASPIYLEKYGEPTSIEDLQKNHKAIHFFSSRTGRNFDWDFVVDD	210
AB341-IK15	ILIPRLRDFHARYPDIDLVIGLNDRPVDLVGEAVDCAIRVGELKDSSLIARRIGTFQCATAASPIYLEKYGEPTSIEDLQKNHKAIHFFSSRTGRNFDWDFVVDD	210
AB342-IK16	ILIPRLRDFHARYPDIDLVIGLNDRPVDLVGEAVDCAIRVGELKDSSLIARRIGTFQCATAASPIYLEKYGEPTSIEDLQKNHKAIHFFSSRTGRNFDWDFVVDD	210
AB343-IK17	ILIPRLRDFHARYPDIDLVIGLNDRPVDLVGEAVDCAIRVGELKDSSLIARRIGTFQCATAASPIYLEKYGEPTSIEDLQKNHKAIHFFSSRTGRNFDWDFVVDD	210
AB345-IK19	ILIPRLRDFHARYPDIDLVIGLNDRPVDLVGEAVDCAIRVGELKDSSLIARRIGTFQCATAASPIYLEKYGEPTSIEDLQKNHKAIHFFSSRTGRNFDWDFVVDD	210
AB346-IK20	ILIPRLRDFHARYPDIDLVIGLNDRPVDLVGEAVDCAIRVGELKDSSLIARRIGTFQCATAASPIYLEKYGEPTSIEDLQKNHKAIHFFSSRTGRNFDWDFVVDD	210
AB347-IK21	ILIPRLRDFHARYPDIDLVIGLNDRPVDLVGEAVDCAIRVGELKDSSLIARRIGTFQCATAASPIYLEKYGEPTSIEDLQKNHKAIHFFSSRTGRNFDWDFVVDD	210
AB351-IK26	ILIPRLRDFHARYPDIDLVIGLNDRPVDLVGEAVDCAIRVGELKDSSLIARRIGTFQCATAASPIYLEKYGEPTSIEDLQKNHKAIHFFSSRTGRNFDWDFVVDD	210
AB352-IK27	ILIPRLRDFHARYPDIDLVIGLNDRPVDLVGEAVDCAIRVGELKDSSLIARRIGTFQCATAASPIYLEKYGEPTSIEDLQKNHKAIHFFSSRTGRNFDWDFVVDD	210
AB353-IK28	ILIPRLRDFHARYPDIDLVIGLNDRPVDLVGEAVDCAIRVGELKDSSLIARRIGTFQCATAASPIYLEKYGEPTSIEDLQKNHKAIHFFSSRTGRNFDWDFVVDD	210
AB354-IK29	ILIPRLRDFHARYPDIDLVIGLNDRPVDLVGEAVDCAIRVGELKDSSLIARRIGTFQCATAASPIYLEKYGEPTSIEDLQKNHKAIHFFSSRTGRNFDWDFVVDD	210
AB214-IK32	ILIPRLRDFHARYPDIDLVIGLNDRPVDLVGEAVDCAIRVGELKDSSLIARRIGTFQCATAASPIYLEKYGEPTSIEDLQKNHKAIHFFSSRTGRNFDWDFVVDD	210
AB215-IK33	ILIPRLRDFHARYPDIDLVIGLNDRPVDLVGEAVDCAIRVGELKDSSLIARRIGTFQCATAASPIYLEKYGEPTSIEDLQKNHKAIHFFSSRTGRNFDWDFVVDD	210
AB216-IK34	ILIPRLRDFHARYPDIDLVIGLNDRPVDLVGEAVDCAIRVGELKDSSLIARRIGTFQCATAASPIYLEKYGEPTSIEDLQKNHKAIHFFSSRTGRNFDWDFVVDD	210
AB218-IK36	ILIPRLRDFHARYPDIDLVIGLNDRPVDLVGEAVDCAIRVGELKDSSLIARRIGTFQCATAASPIYLEKYGEPTSIEDLQKNHKAIHFFSSRTGRNFDWDFVVDD	210
AB219-IK37	ILIPRLRDFHARYPDIDLVIGLNDRPVDLVGEAVDCAIRVGELKDSSLIARRIGTFQCATAASPIYLEKYGEPTSIEDLQKNHKAIHFFSSRTGRNFDWDFVVDD	210
AB220-IK38	ILIPRLRDFHARYPDIDLVIGLNDRPVDLVGEAVDCAIRVGELKDSSLIARRIGTFQCATAASPIYLEKYGEPTSIEDLQKNHKAIHFFSSRTGRNFDWDFVVDD	210
AB223-IK41	ILIPRLRDFHARYPDIDLVIGLNDRPVDLVGEAVDCAIRVGELKDSSLIARRIGTFQCATAASPIYLEKYGEPTSIEDLQKNHKAIHFFSSRTGRNFDWDFVVDD	210
AB224-IK42	ILIPRLRDFHARYPDIDLVIGLNDRPVDLVGEAVDCAIRVGELKDSSLIARRIGTFQCATAASPIYLEKYGEPTSIEDLQKNHKAIHFFSSRTGRNFDWDFVVDD	210
AB052-MST-SNC-8	ILIPRLRDFHARYPDIDLVIGLNDRPVDLVGEAVDCAIRVGELKDSSLIARRIGTFQCATAASPIYLEKYGEPTSIEDLQKNHKAIHFFSSRTGRNFDWDFVVDD	210
AB421-MST-SNC-9	ILIPRLRDFHARYPDIDLVIGLNDRPVDLVGEAVDCAIRVGELKDSSLIARRIGTFQCATAASPIYLEKYGEPTSIEDLQKNHKAIHFFSSRTGRNFDWDFVVDD	210
AB422-MST-SNC-24	ILIPRLRDFHARYPDIDLVIGLNDRPVDLVGEAVDCAIRVGELKDSSLIARRIGTFQCATAASPIYLEKYGEPTSIEDLQKNHKAIHFFSSRTGRNFDWDFVVDD	210
AB423-MST-SNC-253	ILIPRLRDFHARYPDIDLVIGLNDRPVDLVGEAVDCAIRVGELKDSSLIARRIGTFQCATAASPIYLEKYGEPTSIEDLQKNHKAIHFFSSRTGRNFDWDFVVDD	210
AB424-AcS9	ILIPRLRDFHARYPDIDLVIGLNDRPVDLVGEAVDCAIRVGELKDSSLIARRIGTFQCATAASPIYLEKYGEPTSIEDLQKNHKAIHFFSSRTGRNFDWDFVVDD	210
AB425-AcS17	ILIPRLRDFHARYPDIDLVIGLNDRPVDLVGEAVDCAIRVGELKDSSLIARRIGTFQCATAASPIYLEKYGEPTSIEDLQKNHKAIHFFSSRTGRNFDWDFVVDD	210
AB426-AcS18	ILIPRLRDFHARYPDIDLVIGLNDRPVDLVGEAVDCAIRVGELKDSSLIARRIGTFQCATAASPIYLEKYGEPTSIEDLQKNHKAIHFFSSRTGRNFDWDFVVDD	210
AB427-AcS19	ILIPRLRDFHARYPDIDLVIGLNDRPVDLVGEAVDCAIRVGELKDSSLIARRIGTFQCATAASPIYLEKYGEPTSIEDLQKNHKAIHFFSSRTGRNFDWDFVVDD	210
AB428-AcS20	ILIPRLRDFHARYPDIDLVIGLNDRPVDLVGEAVDCAIRVGELKDSSLIARRIGTFQCATAASPIYLEKYGEPTSIEDLQKNHKAIHFFSSRTGRNFDWDFVVDD	210
AB429-AcS27	ILIPRLRDFHARYPDIDLVIGLNDRPVDLVGEAVDCAIRVGELKDSSLIARRIGTFQCATAASPIYLEKYGEPTSIEDLQKNHKAIHFFSSRTGRNFDWDFVVDD	210
AB420-MST-SNC-5	ILIPRLRDFHARYPDIDLVIGLNDRPVDLVGEAVDCAIRVGELKDSSLIARRIGTFQCATAASPIYLEKYGEPTSIEDLQKNHKAIHFFSSRTGRNFDWDFVVDD	210
consensus	!!**************	

Figure 3 – AdeL

```
ATCC17978           LIKSVSVRGRVSVNDGDAYIDLALQGFGIIQGPRYMLTNHLESGLLKEVLPQWTPAPMPISAVYLQNRHLSLKVKVFVDWVAELFAGCPLLGGTALPFDQKCEFA   315
AB030               .........................................................................................................   196
AB046               LIKSVSVRGRVSVNDGDAYIDLALQGFGIIQGPRYMLTNHLESGLLKEVLPQWTPAPMPISAVYLQNRHLSLKVKVFVDWVAELFAGCPLLGGTALPFDQKCEFA   315
AB337-IK11          LIKSVSVRGRVSVNDGDAYIDLALQGFGIIQGPRYMLTNHLESGLLKEVLPQWTPAPMPISAVYLQNRHLSLKVKVFVDWVAELFAGCPLLGGTALPFDQKCEFA   315
AB338-IK12          LIKSVSVRGRVSVNDGDAYIDLALQGFGIIQGPRYMLTNHLESGLLKEVLPQWTPAPMPISAVYLQNRHLSLKVKVFVDWVAELFAGCPLLGGTALPFDQKCEFA   315
AB339-IK13          LIKSVSVRGRVSVNDGDAYIDLALQGFGIIQGPRYMLTNHLESGLLKEVLPQWTPAPMPISAVYLQNRHLSLKVKVFVDWVAELFAGCPLLGGTALPFDQKCEFA   315
AB340-IK14          LIKSVSVRGRVSVNDGDAYIDLALQGFGIIQGPRYMLTNHLESGLLKEVLPQWTPAPMPISAVYLQNRHLSLKVKVFVDWVAELFAGCPLLGGTALPFDQKCEFA   315
AB341-IK15          LIKSVSVRGRVSVNDGDAYIDLALQGFGIIQGPRYMLTNHLESGLLKEVLPQWTPAPMPISAVYLQNRHLSLKVKVFVDWVAELFAGCPLLGGTALPFDQKCEFA   315
AB342-IK16          LIKSVSVRGRVSVNDGDAYIDLALQGFGIIQGPRYMLTNHLESGLLKEVLPQWTPAPMPISAVYLQNRHLSLKVKVFVDWVAELFAGCPLLGGTALPFDQKCEFA   315
AB343-IK17          LIKSVSVRGRVSVNDGDAYIDLALQGFGIIQGPRYMLTNHLESGLLKEVLPQWTPAPMPISAVYLQNRHLSLKVKVFVDWVAELFAGCPLLGGTALPFDQKCEFA   315
AB345-IK19          LIKSVSVRGRVSVNDGDAYIDLALQGFGIIQGPRYMLTNHLESGLLKEVLPQWTPAPMPISAVYLQNRHLSLKVKVFVDWVAELFAGCPLLGGTALPFDQKCEFA   315
AB346-IK20          LIKSVSVRGRVSVNDGDAYIDLALQGFGIIQGPRYMLTNHLESGLLKEVLPQWTPAPMPISAVYLQNRHLSLKVKVFVDWVAELFAGCPLLGGTALPFDQKCEFA   315
AB347-IK21          LIKSVSVRGRVSVNDGDAYIDLALQGFGIIQGPRYMLTNHLESGLLKEVLPQWTPAPMPISAVYLQNRHLSLKVKVFVDWVAELFAGCPLLGGTALPFDQKCEFA   315
AB351-IK26          LIKSVSVRGRVSVNDGDAYIDLALQGFGIIQGPRYMLTNHLESGLLKEVLPQWTPAPMPISAVYLQNRHLSLKVKVFVDWVAELFAGCPLLGGTALPFDQKCEFA   315
AB352-IK27          LIKSVSVRGRVSVNDGDAYIDLALQGFGIIQGPRYMLTNHLESGLLKEVLPQWTPAPMPISAVYLQNRHLSLKVKVFVDWVAELFAGCPLLGGTALPFDQKCEFA   315
AB353-IK28          LIKSVSVRGRVSVNDGDAYIDLALQGFGIIQGPRYMLTNHLESGLLKEVLPQWTPAPMPISAVYLQNRHLSLKVKVFVDWVAELFAGCPLLGGTALPFDQKCEFA   315
AB354-IK29          LIKSVSVRGRVSVNDGDAYIDLALQGFGIIQGPRYMLTNHLESGLLKEVLPQWTPAPMPISAVYLQNRHLSLKVKVFVDWVAELFAGCPLLGGTALPFDQKCEFA   315
AB214-IK32          LIKSVSVRGRVSVNDGDAYIDLALQGFGIIQGPRYMLTNHLESGLLKEVLPQWTPAPMPISAVYLQNRHLSLKVKVFVDWVAELFAGCPLLGGTALPFDQKCEFA   315
AB215-IK33          LIKSVSVRGRVSVNDGDAYIDLALQGFGIIQGPRYMLTNHLESGLLKEVLPQWTPAPMPISAVYLQNRHLSLKVKVFVDWVAELFAGCPLLGGTALPFDQKCEFA   315
AB216-IK34          LIKSVSVRGRVSVNDGDAYIDLALQGFGIIQGPRYMLTNHLESGLLKEVLPQWTPAPMPISAVYLQNRHLSLKVKVFVDWVAELFAGCPLLGGTALPFDQKCEFA   315
AB218-IK36          LIKSVSVRGRVSVNDGDAYIDLALQGFGIIQGPRYMLTNHLESGLLKEVLPQWTPAPMPISAVYLQNRHLSLKVKVFVDWVAELFAGCPLLGGTALPFDQKCEFA   315
AB219-IK37          LIKSVSVRGRVSVNDGDAYIDLALQGFGIIQGPRYMLTNHLESGLLKEVLPQWTPAPMPISAVYLQNRHLSLKVKVFVDWVAELFAGCPLLGGTALPFDQKCEFA   315
AB220-IK38          LIKSVSVRGRVSVNDGDAYIDLALQGFGIIQGPRYMLTNHLESGLLKEVLPQWTPAPMPISAVYLQNRHLSLKVKVFVDWVAELFAGCPLLGGTALPFDQKCEFA   315
AB223-IK41          LIKSVSVRGRVSVNDGDAYIDLALQGFGIIQGPRYMLTNHLESGLLKEVLPQWTPAPMPISAVYLQNRHLSLKVKVFVDWVAELFAGCPLLGGTALPFDQKCEFA   315
AB224-IK42          LIKSVSVRGRVSVNDGDAYIDLALQGFGIIQGPRYMLTNHLESGLLKEVLPQWTPAPMPISAVYLQNRHLSLKVKVFVDWVAELFAGCPLLGGTALPFDQKCEFA   315
AB052-MST-SNC-8     LIKSVSVRGRVSVNDGDAYIDLALQGFGIIQGPRYMLTNHLESGLLKEVLPQWTPAPMPISAVYLQNRHLSLKVKVFVDWVAELFAGCPLLGGTALPFDQKCEFA   315
AB421-MST-SNC-9     LIKSVSVRGRVSVNDGDAYIDLALQGFGIIQGPRYMLTNHLESGLLKEVLPQWTPAPMPISAVYLQNRHLSLKVKVFVDWVAELFAGCPLLGGTALPFDQKCEFA   315
AB422-MST-SNC-24    LIKSVSVRGRVSVNDGDAYIDLALQGFGIIQGPRYMLTNHLESGLLKEVLPQWTPAPMPISAVYLQNRHLSLKVKVFVDWVAELFAGCPLLGGTALPFDQKCEFA   315
AB423-MST-SNC-253   LIKSVSVRGRVSVNDGDAYIDLALQGFGIIQGPRYMLTNHLESGLLKEVLPQWTPAPMPISAVYLQNRHLSLKVKVFVDWVAELFAGCPLLGGTALPFDQKCEFA   315
AB424-AcS9          LIKSVSVRGRVSVNDGDAYIDLALQGFGIIQGPRYMLTNHLESGLLKEVLPQWTPAPMPISAVYLQNRHLSLKVKVFVDWVAELFAGCPLLGGTALPFDQKCEFA   315
AB425-AcS17         LIKSVSVRGRVSVNDGDAYIDLALQGFGIIQGPRYMLTNHLESGLLKEVLPQWTPAPMPISAVYLQNRHLSLKVKVFVDWVAELFAGCPLLGGTALPFDQKCEFA   315
AB426-AcS18         LIKSVSVRGRVSVNDGDAYIDLALQGFGIIQGPRYMLTNHLESGLLKEVLPQWTPAPMPISAVYLQNRHLSLKVKVFVDWVAELFAGCPLLGGTALPFDQKCEFA   315
AB427-AcS19         LIKSVSVRGRVSVNDGDAYIDLALQGFGIIQGPRYMLTNHLESGLLKEVLPQWTPAPMPISAVYLQNRHLSLKVKVFVDWVAELFAGCPLLGGTALPFDQKCEFA   315
AB428-AcS20         LIKSVSVRGRVSVNDGDAYIDLALQGFGIIQGPRYMLTNHLESGLLKEVLPQWTPAPMPISAVYLQNRHLSLKVKVFVDWVAELFAGCPLLGGTALPFDQKCEFA   315
AB429-AcS27         LIKSVSVRGRVSVNDGDAYIDLALQGFGIIQGPRYMLTNHLESGLLKEVLPQWTPAPMPISAVYLQNRHLSLKVKVFVDWVAELFAGCPLLGGTALPFDQKCEFA   315
AB420-MST-SNC-5     LIKSVSVRGRVSVNDGDAYIDLALQGFGIIQGPRYMLTNHLESGLLKEVLPQWTPAPMPISAVYLQNRHLSLKVKVFVDWVAELFAGCPLLGGTALPFDQKCEFA   315
consensus           *********************************************************************************************************
```

Figure 4 – AdeL

```
ATCC17978            CDKETGHEYTIRTLVEQHNIAEAYTLKT  343
AB030                ............................  196
AB046                CDKETGHEYTIRTLVEQHNIAEAYTLKT  343
AB337-IK11           CDKETGHEYTIRTLVEQHNIAEAYTLKT  343
AB338-IK12           CDKETGHEYTIRTLVEQHNIAEAYTLKT  343
AB339-IK13           CDKETGHEYTIRTLVEQHNIAEAYTLKT  343
AB340-IK14           CDKETGHEYTIRTLVEQHNIAEAYTLKT  343
AB341-IK15           CDKETGHEYTIRTLVEQHNIAEAYTLKT  343
AB342-IK16           CDKETGHEYTIRTLVEQHNIAEAYTLKT  343
AB343-IK17           CDKETGHEYTIRTLVEQHNIAEAYTLKT  343
AB345-IK19           CDKETGHEYTIRTLVEQHNIAEAYTLKT  343
AB346-IK20           CDKETGHEYTIRTLVEQHNIAEAYTLKT  343
AB347-IK21           CDKETGHEYTIRTLVEQHNIAEAYTLKT  343
AB351-IK26           CDKETGHEYTIRTLVEQHNIAEAYTLKT  343
AB352-IK27           CDKETGHEYTIRTLVEQHNIAEAYTLKT  343
AB353-IK28           CDKETGHEYTIRTLVEQHNIAEAYTLKT  343
AB354-IK29           CDKETGHEYTIRTLVEQHNIAEAYTLKT  343
AB214-IK32           CDKETGHEYTIRTLVEQHNIAEAYTLKT  343
AB215-IK33           CDKETGHEYTIRTLVEQHNIAEAYTLKT  343
AB216-IK34           CDKETGHEYTIRTLVEQHNIAEAYTLKT  343
AB218-IK36           CDKETGHEYTIRTLVEQHNIAEAYTLKT  343
AB219-IK37           CDKETGHEYTIRTLVEQHNIAEAYTLKT  343
AB220-IK38           CDKETGHEYTIRTLVEQHNIAEAYTLKT  343
AB223-IK41           CDKETGHEYTIRTLVEQHNIAEAYTLKT  343
AB224-IK42           CDKETGHEYTIRTLVEQHNIAEAYTLKT  343
AB052-MST-SNC-8      CDKETGHEYTIRTLVEQHNIAEAYTLKT  343
AB421-MST-SNC-9      CDKETGHEYTIRTLVEQHNIAEAYTLKT  343
AB422-MST-SNC-24     CDKETGHEYTIRTLVEQHNIAEAYTLKT  343
AB423-MST-SNC-253    CDKETGHEYTIRTLVEQHNIAEAYTLKT  343
AB424-AcS9           CDKETGHEYTIRTLVEQHNIAEAYTLKT  343
AB425-AcS17          CDKETGHEYTIRTLVEQHNIAEAYTLKT  343
AB426-AcS18          CDKETGHEYTIRTLVEQHNIAEAYTLKT  343
AB427-AcS19          CDKETGHEYTIRTLVEQHNIAEAYTLKT  343
AB428-AcS20          CDKETGHEYTIRTLVEQHNIAEAYTLKT  343
AB429-AcS27          CDKETGHEYTIRTLVEQHNIAEAYTLKT  343
AB420-MST-SNC-5      CDKETGHEYTIRTLVEQHNIAEAYTLKT  343
consensus            ****************************
```

X non-conserved

X ≥50% conserved

Figure 4a – Motility - hospital

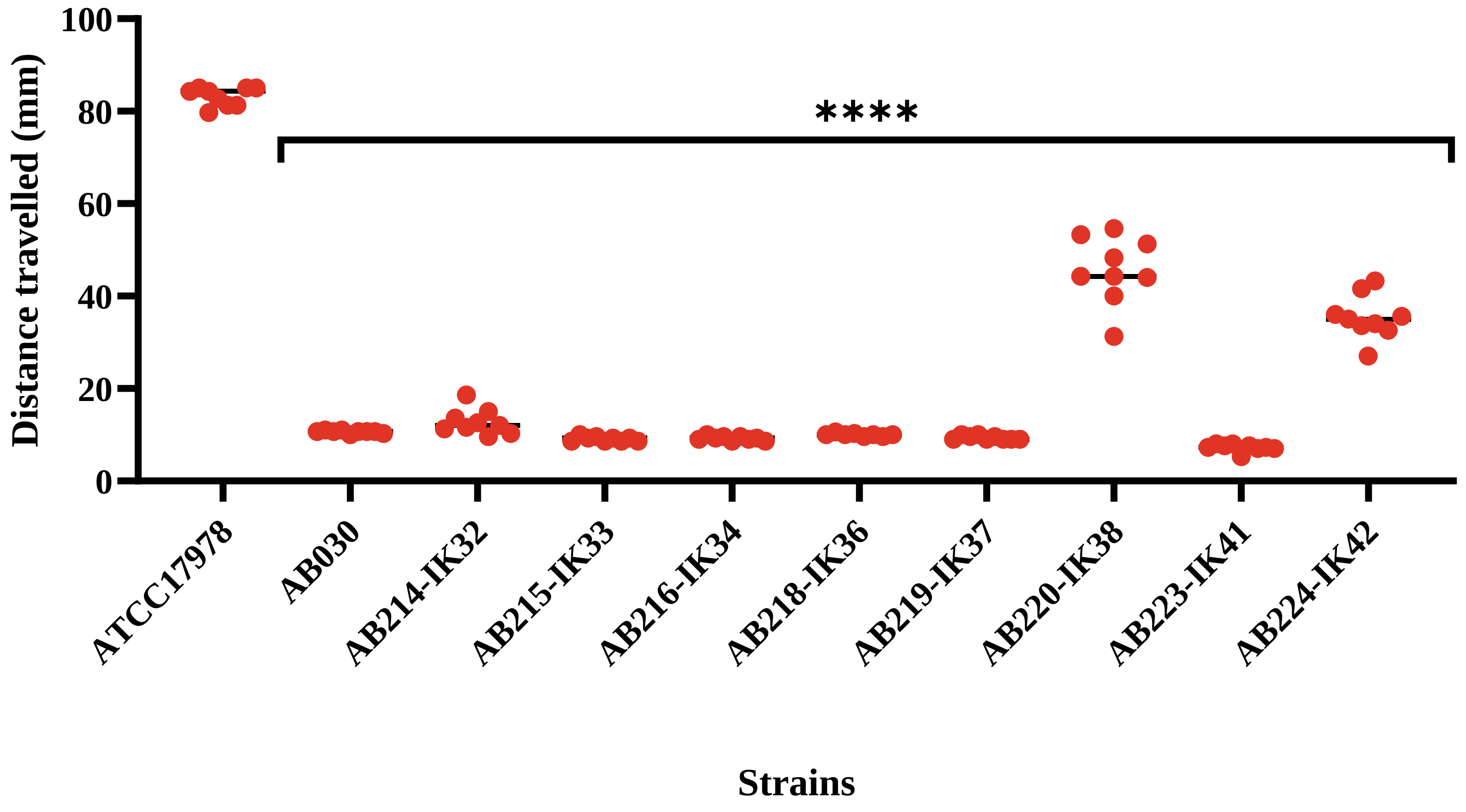

Figure 4b – Motility – Tank milk

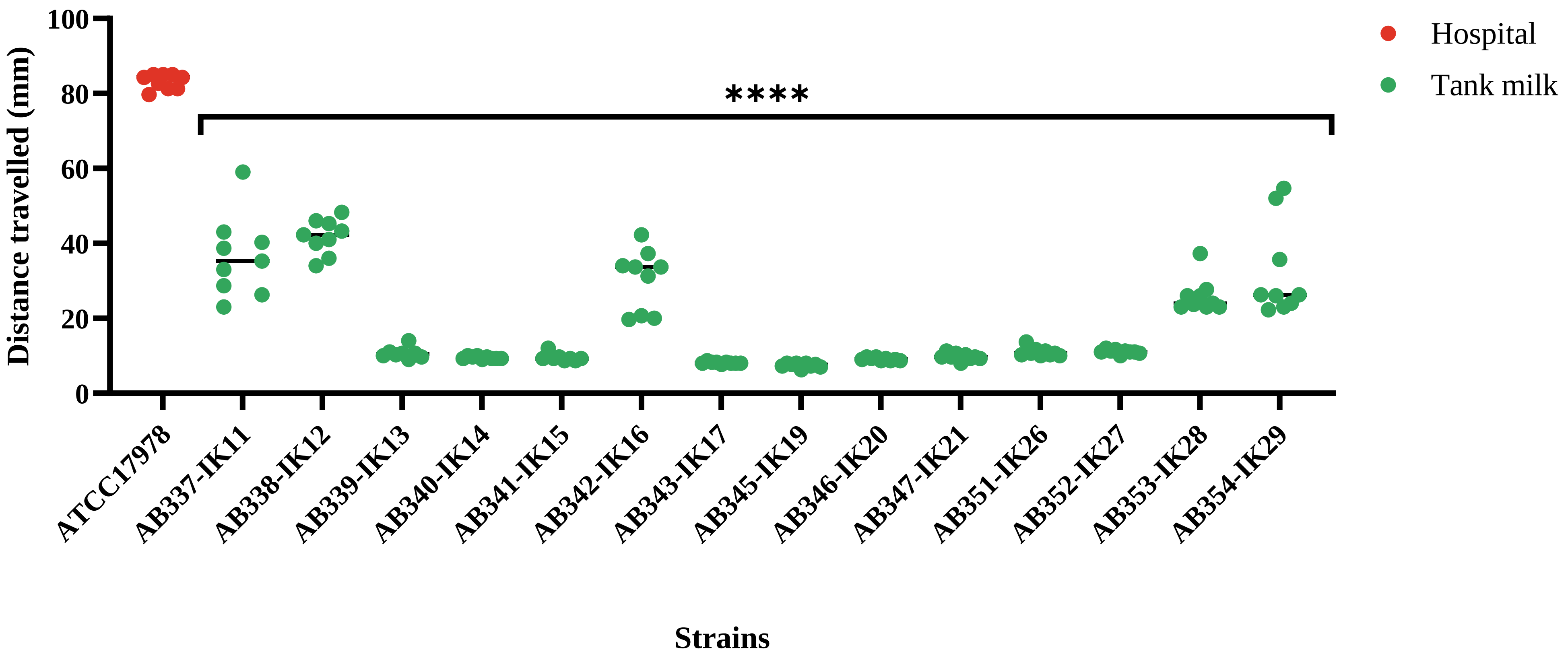

Figure 4c – Motility - Stream and WWE

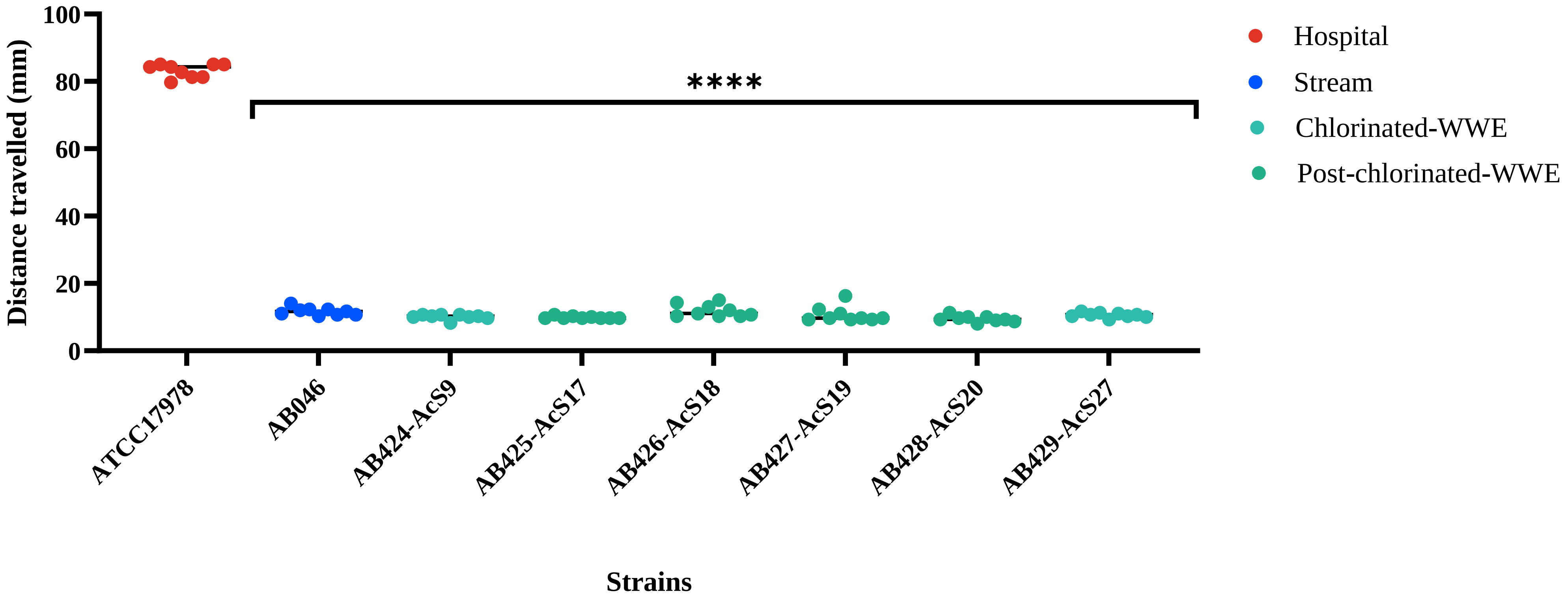

Figure 4d – Motility – Agricultural Surface Water

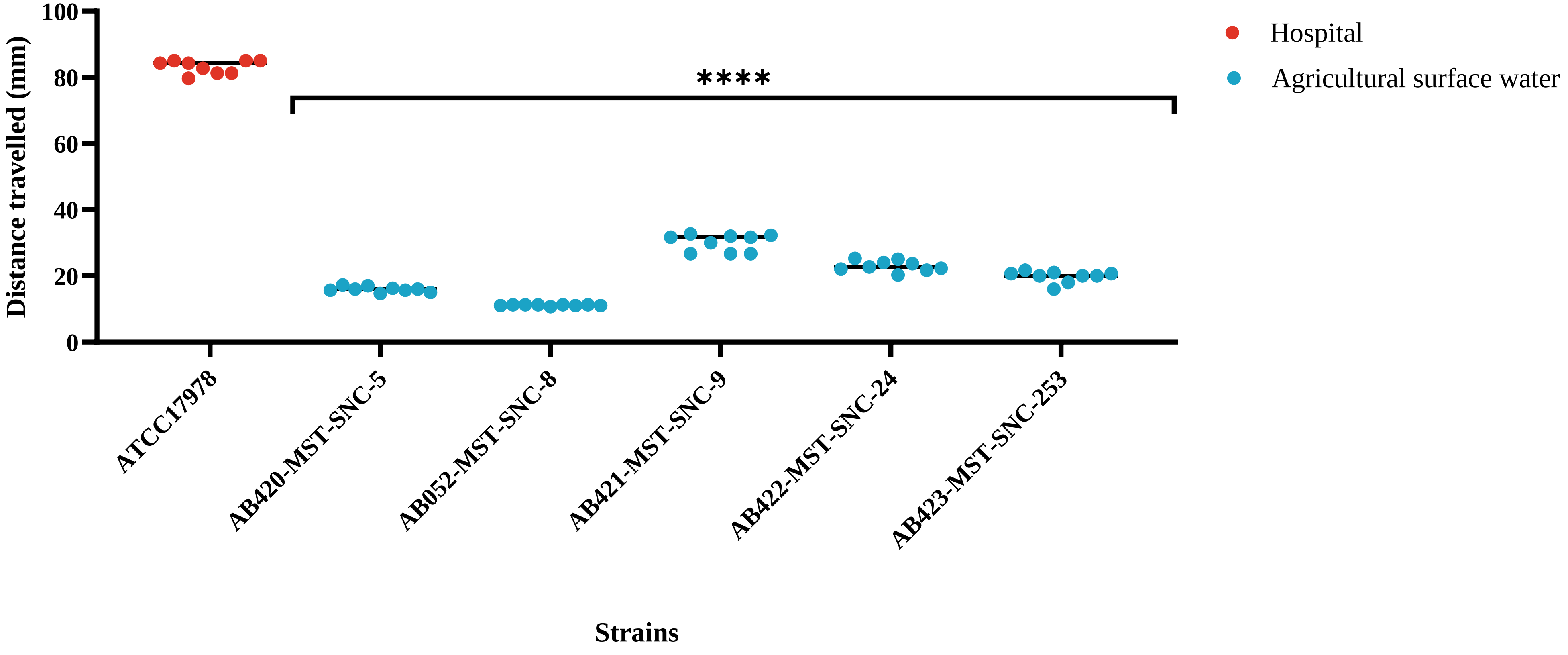

Figure 5a: Biofilm Formation - Hospital

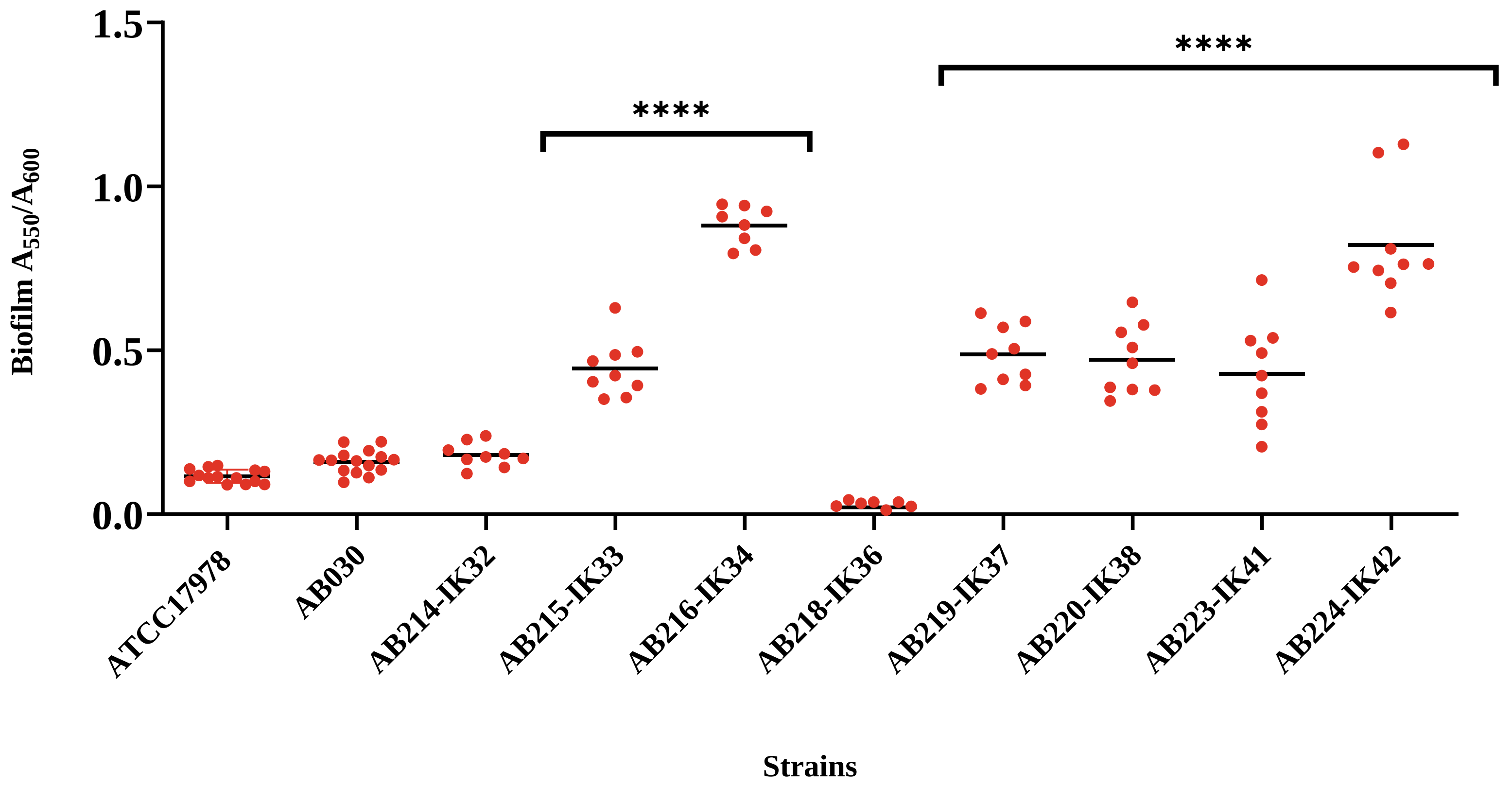

Figure 5b: Biofilm Formation – Tank milk

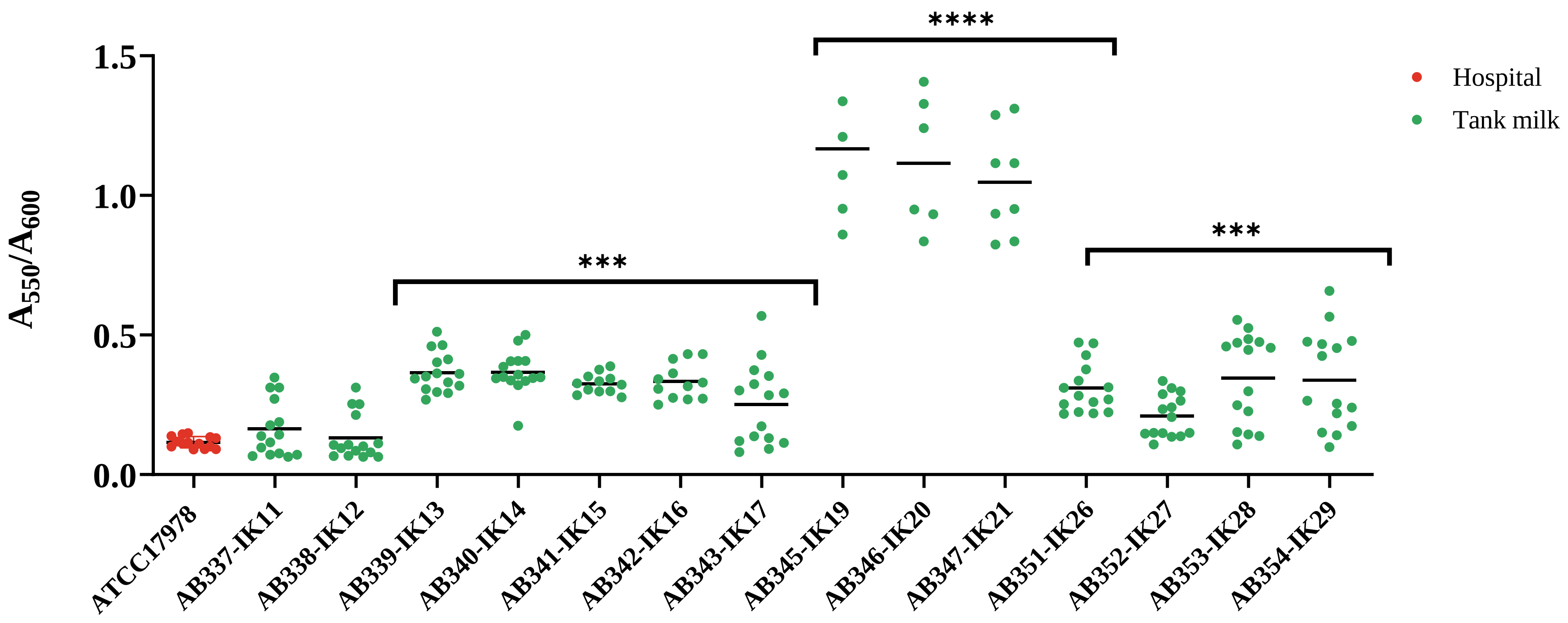

Figure 5c: Biofilm Formation – Stream and WWTP

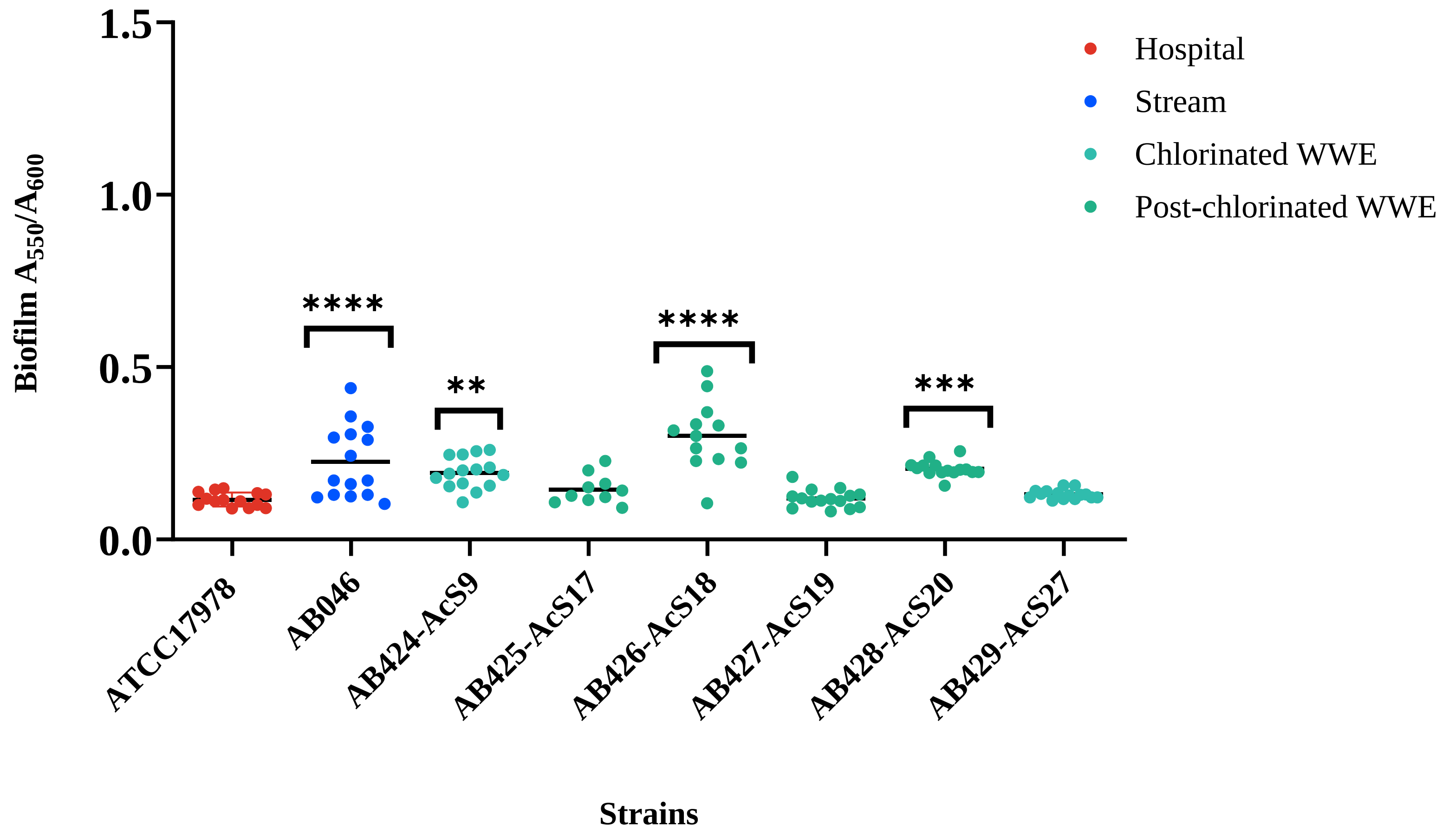

Figure 5d: Biofilm Formation – Agricultural Surface water

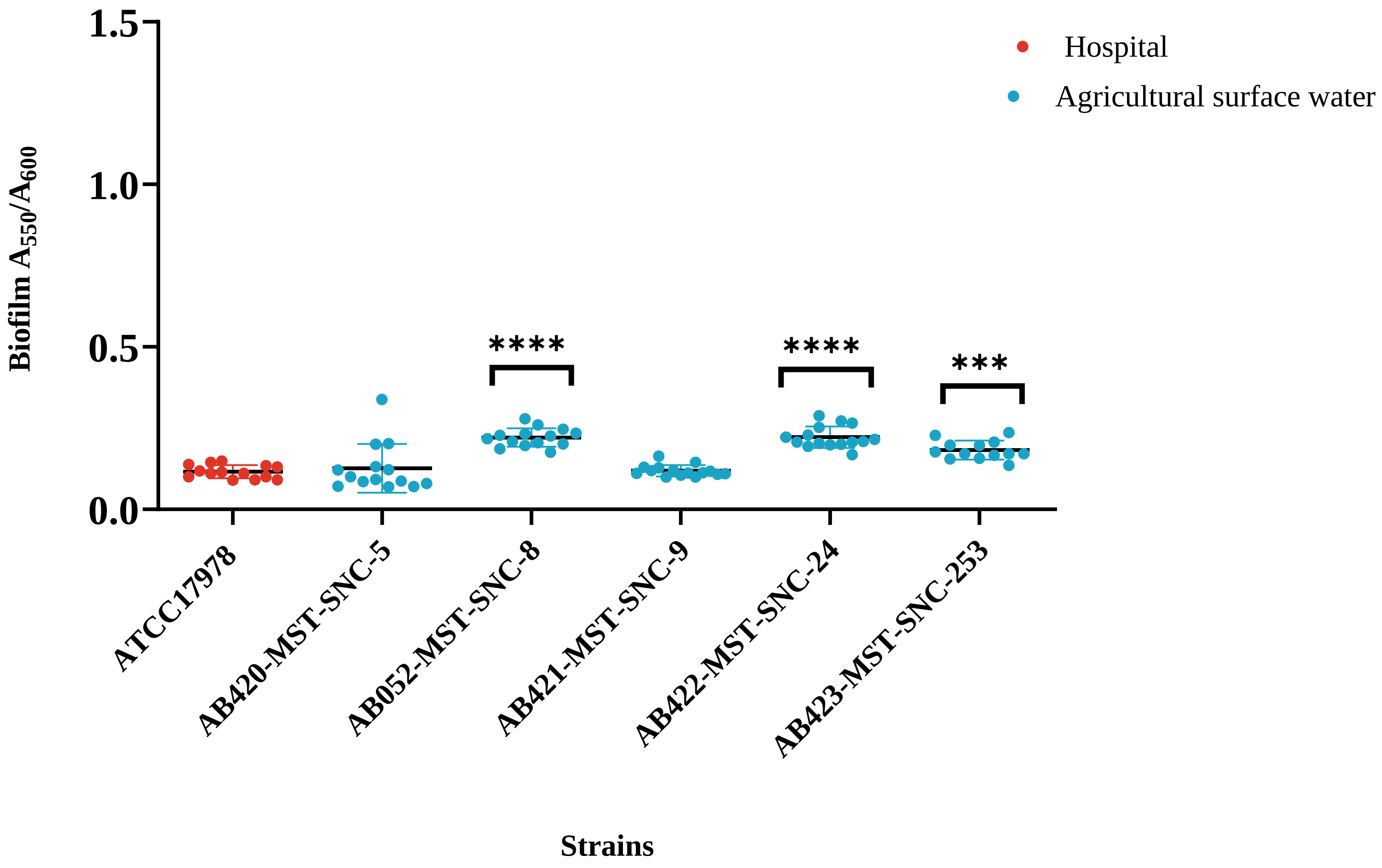

Figure 6: Spearman's Correlation Analysis of Motility and Biofilm Formation

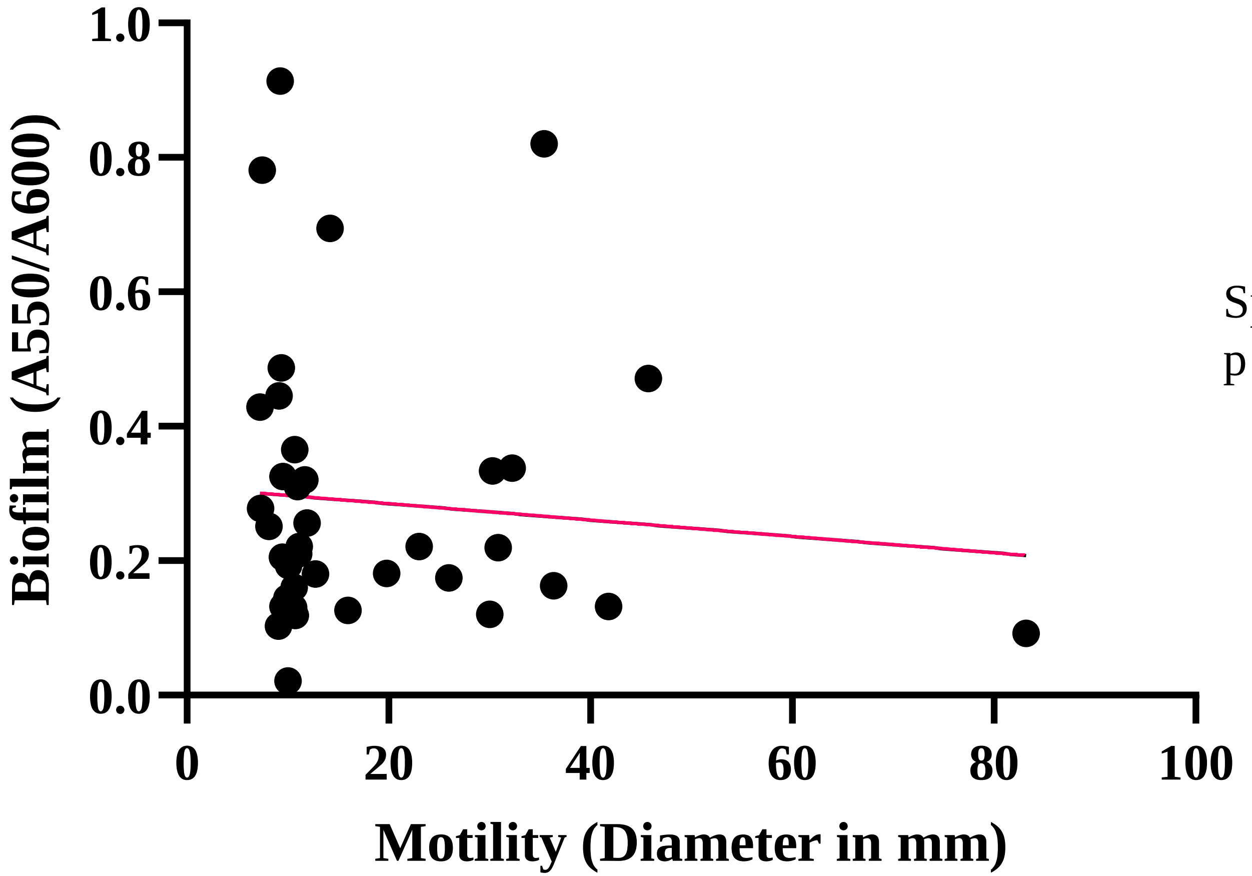

Spearman correlation R = -0.1382
p = 0.4016
