# Supplementary material for: Phylogenomic and phenotypic analyses highlight the diversity of antibiotic resistance and virulence in both human and non-human Acinetobacter baumannii
Source: mSphere. 2024 Mar 5;9(3):e00741-23. doi: 10.1128/msphere.00741-23 (PMC10964423; doi:10.1128/msphere.00741-23)
Supplement: Supplemental Text — Supplemental methods and figure legends. [file msphere.00741-23-s0002.docx]

Supplemental Methods:

**Whole Genome Sequencing**

Genomic DNA was isolated using the DNAeasy UltraClean microbial kit (Qiagen, MD, USA) from a purified colony of each strain according to the manufacturer’s instructions. Sequence libraries were prepared and pooled using the DNA prep and the NextSeq 500 mid output reagent kits (Illumina, CA, USA). Illumina NextSeq 500 platform, at the AAFC-ORDC, was used for whole-genome sequencing and *de novo* assembled using SPAdes v. 3.12.0 (1). Completeness and quality of genome assembly was performed using QUAST v. 5.0.2 (2) and CheckM v. 1.0.11 (3) with a 95% completeness and equal or less than 5% contamination accepted. AB344-IK18 and AB429-AcS27 failed the initial CheckM examination. Therefore, they were also prepared using the LSK-110 ligation sequencing kit from Oxford Nanopore technologies (ONT) and then run using a ONT MinIon. These genomes were hybrid assembled using Unicycler v. 1.2 (4). Accession numbers: Bioproject PRJNA819071, and Biosamples SAMN26898552 - SAMN26898587.

**Novel Sequence Type and Clonal Complex Assignment**

All novel sequence type (ST) profiles were uploaded to pubMLST.org to await ST assignment. Genomes for the novel STs were also uploaded as well. In order to determine Clonal Complex (CC) assignment, as per Gaiarsa *et al* 2019 (5), Phyloviz (6) was used to facilitate the goeBURST algorithm of analysis (7). CC assignment was determined based on those STs that share identical alleles at six of seven loci and the CC was named according to the founder ST. If no founder ST was determined, then that ST remains unassigned to current CCs.

**Promoter and Regulator Analysis**

For promoter analysis, 500 bp upstream of the gene of interest was extracted from the reference genome, ATCC17978 (Accession: NZ_CP018664.1). BLASTN was performed for *adeI, adeA,* and *adeF* to determine homology of promoter regions. For regulator analysis, the amino acid sequences of AdeN, AdeR, BaeR and AdeL were extracted from ATCC17978 (Accession: NZ_CP018664.1). Using tBLASTn, percent identity was determined. Nucleotide and amino acid sequences were aligned using ClustalOmega (8) and sequence logo and formatting were performed using the msaprettyprint package (9) in R Studio.

**Biofilm Formation**

Overnight cultures of each strain were measured using A_600_. Cultures were standardized to 1.0 and then 150 µL inoculated in flat bottom polystyrene plates. Incubated at 37ºC for 48 hours followed by measurement of A_600_. Removal of planktonic cells was done via washing with sterile mQH_2_O. Staining of the biofilm with 0.1% crystal violet was performed for 30 minutes followed by 3 washes with sterile mQH_2_O. Solubilization of the stained biofilm was done using 30% acetic acid for 30 minutes after which A_550_ was determined. Biofilm biomass values were standardized according to the cell biomass present prior to straining. Five technical replicates were performed with at least three biological replicates for each strain. Correlation analysis was performed by plotting the average A_550_/A_600_ value for each strain on the Y axis and the average distance travelled for motility on the X. Spearman’s correlation was performed in GraphPad Prism 10.0.0.

**Motility**

Overnight cultures of each strain were measured at A_600_. Cultures were standardized to A_600_ of 1.0. Then 3 µL was stab inoculated into the centre of semi-solid minimal media made of 0.5% tryptone (Becton, Dickenson and Company, MD, USA), 0.3% agarose (BioBasic Canada Inc, Markham, ON, Canada), and 0.5% NaCl (BioShop Canada Inc., Burlington, ON, Canada) and incubated at 37ºC for 18 hours. Three measurements around the diameter of distance travelled were recorded. At least three biological replicates with three technical replicates for each strain were performed. Correlation analysis was performed as mentioned in the biofilm formation section of the methods.

Supplemental Figure and Table Legends

**Supplemental Figure 1:** Alignment of AdeN dimerization domain (amino acid 92 – 214) from all *A. baumannii* isolates in this study. The consensus sequence has been noted on the top of the alignment. Stars at the bottom indicate 100% conservation. Exclamation marks indicate at least one amino acid is not conserved. The blue colour indicates ≥50% conservation between all sequences. Strain AB428-AcS20 is truncated at E126.

**Supplemental Figure 2:** Alignments of AdeR. The consensus sequence has been noted on the top of the alignment. Stars at the bottom indicate 100% conservation. Exclamation marks indicate at least one amino acid is not conserved. The blue colour indicates ≥50% conservation between all sequences. **A:** Alignment of AdeR DNA binding domain (amino acid 230 – 247) from all *A. baumannii* isolates in this study. Strains AB339-IK14, AB340-IK15, AB341-IK16 and AB220-IK38 show truncation of AdeR at P240. **B:** Complete AdeR alignment of AB339-IK14, AB340-IK15, AB341-IK16 and AB220-IK38. AdeR in these strains is 100% conserved.

**Supplemental Figure 3:** Alignment of AdeL (amino acid 196 – 302) from all *A. baumannii* isolates in this study. The consensus sequence has been noted on the top of the alignment. Stars at the bottom indicate 100% conservation. Exclamation marks indicate at least one amino acid is not conserved. The blue colour indicates ≥50% conservation between all sequences. Strain AB030 is truncated at T196.

**Supplemental Figure 4:** Motility evaluation of all *A. baumannii* isolates. Each strain is displayed on the x axis and distance travelled is plotted on the y axis. The colour indicated signifies the isolation source of each strain. Significance was calculated relative to ATCC17978 using a One-Way ANOVA in GraphPad 10.0.0 where **** is p<0.0001. **A:** Motility of hospital isolates. **B:** Motility of tank milk isolates. **C:** Motility of isolates from stream and various waste-water treatment plant (WWE). **D:** Motility of agricultural surface water isolates.

**Supplemental Figure 5:** Biofilm formation evaluation of all *A. baumannii* isolates. Each strain is displayed on the x axis and biofilm biomass (measured as amount of biofilm stained by crystal violet and measured at A_550_) normalized to the initial absorbance of cells at A_600_ is shown on the y axis. The colour indicated signifies the isolation source of each strain. Significance was calculated relative to ATCC17978 in GraphPad 10.0.0 where **** is p<0.0001 and *** is p ≥ 0.001. **A:** Biofilm formation of hospital isolates. **B:** Biofilm evaluation of tank milk isolates. **C:** Biofilm formation of isolates from stream and various waste-water treatment plant (WWE). **D:** Biofilm evaluation of agricultural surface water isolates.

**Supplemental Figure 6:** Biofilm and motility correlation analysis. The average distance travelled on motility plates was plotted against biofilm biomass for each isolate. GraphPad 10.0.0 was used to perform a Spearman correlation analysis on the non-linear data.

**Supplemental Table 1:** Strains used in this study. All strains are noted and their isolation source, geographic origin, isolation media and genome assembly accession number are assigned.

**Supplemental Table 3:** Novel sequence type and clonal complex assignments.

**Supplemental Table 5:** Primers used in this study. All oligonucleotides used for RT-qPCR are listed here.

Supplemental References

1. Bankevich A, Nurk S, Antipov D, Gurevich AA, Dvorkin M, Kulikov AS, Lesin VM, Nikolenko SI, Pham S, Prjibelski AD, Pyshkin AV, Sirotkin AV, Vyahhi N, Tesler G, Alekseyev MA, Pevzner PA. 2012. SPAdes: a new genome assembly algorithm and its applications to single-cell sequencing. J Comput Biol 19:455-77.

2. Gurevich A, Saveliev V, Vyahhi N, Tesler G. 2013. QUAST: quality assessment tool for genome assemblies. Bioinformatics 29:1072-1075.

3. Parks DH, Imelfort M, Skennerton CT, Hugenholtz P, Tyson GW. 2015. CheckM: assessing the quality of microbial genomes recovered from isolates, single cells, and metagenomes. Genome Res 25:1043-55.

4. Wick RR, Judd LM, Gorrie CL, Holt KE. 2017. Completing bacterial genome assemblies with multiplex MinION sequencing. Microbial Genomics 3.

5. Gaiarsa S, Batisti Biffignandi G, Esposito EP, Castelli M, Jolley KA, Brisse S, Sassera D, Zarrilli R. 2019. Comparative Analysis of the Two Acinetobacter baumannii Multilocus Sequence Typing (MLST) Schemes. Frontiers in Microbiology 10.

6. Nascimento M, Sousa A, Ramirez M, Francisco AP, Carriço JA, Vaz C. 2016. PHYLOViZ 2.0: providing scalable data integration and visualization for multiple phylogenetic inference methods. Bioinformatics 33:128-129.

7. Francisco AP, Bugalho M, Ramirez M, Carriço JA. 2009. Global optimal eBURST analysis of multilocus typing data using a graphic matroid approach. BMC Bioinformatics 10:152.

8. Sievers F, Wilm A, Dineen D, Gibson TJ, Karplus K, Li W, Lopez R, McWilliam H, Remmert M, Söding J, Thompson JD, Higgins DG. 2011. Fast, scalable generation of high‐quality protein multiple sequence alignments using Clustal Omega. Molecular Systems Biology 7:539.

9. Bodenhofer U, Bonatesta E, Horejš-Kainrath C, Hochreiter S. 2015. msa: an R package for multiple sequence alignment. Bioinformatics 31:3997-3999.
