# Supplementary material for: Phylogenomic and phenotypic analyses highlight the diversity of antibiotic resistance and virulence in both human and non-human Acinetobacter baumannii
Source: mSphere. 2024 Mar 5;9(3):e00741-23. doi: 10.1128/msphere.00741-23 (PMC10964423; doi:10.1128/msphere.00741-23)
Supplement: Supplemental Tables — Tables S1, S3, and S5. [file msphere.00741-23-s0003.docx]

Supplemental Tables

Supplemental Table 1: Collection of 36 *A. baumannii* isolates. Metadata for each assembly submitted to Genbank.

| Strain | Isolation Source | Geographic Origin | Isolation Media | Accession |
| --- | --- | --- | --- | --- |
| ATCC17978 | Hospital | France | NA* | NZ_CP018664.1 |
| AB030 | Hospital | Winnipeg, Canada | CHR | NZ_CP009257.1 |
| AB046 | Stream | Ottawa, Canada | Karmali media | NZ_CP037872.1 |
| AB337-IK11 | Tank milk | Hesse, Germany | Nutrient agar /CHR | SAMN26898552 |
| AB338-IK12 | Tank milk | Hesse, Germany | Nutrient agar /CHR | SAMN26898553 |
| AB339-IK13 | Tank milk | Hesse, Germany | Nutrient agar /CHR | SAMN26898554 |
| AB340-IK14 | Tank milk | Hesse, Germany | Nutrient agar /CHR | SAMN26898555 |
| AB341-IK15 | Tank milk | Hesse, Germany | Nutrient agar /CHR | SAMN26898556 |
| AB342-IK16 | Tank milk | Hesse, Germany | Nutrient agar /CHR | SAMN26898557 |
| AB343-IK17 | Tank milk | Bogor, Indonesia | Nutrient agar /CHR | SAMN26898558 |
| AB345-IK19 | Tank milk | Bogor, Indonesia | Nutrient agar /CHR | SAMN26898560 |
| AB346-IK20 | Tank milk | Bogor, Indonesia | Nutrient agar /CHR | SAMN26898561 |
| AB347-IK21 | Tank milk | Bogor, Indonesia | Nutrient agar /CHR | SAMN26898562 |
| AB351-IK26 | Tank milk | Bogor, Indonesia | Nutrient agar /CHR | SAMN26898563 |
| AB352-IK27 | Tank milk | Bogor, Indonesia | Nutrient agar /CHR | SAMN26898564 |
| AB353-IK28 | Tank milk | Bogor, Indonesia | Nutrient agar /CHR | SAMN26898565 |
| AB354-IK29 | Tank milk | Bogor, Indonesia | Nutrient agar /CHR | SAMN26898566 |
| AB214-IK32 | Hospital | Ottawa, Canada | TSA (5% SB)/CHR | SAMN26898567 |
| AB215-IK33 | Hospital | Ottawa, Canada | TSA (5% SB)/CHR | SAMN26898568 |
| AB216-IK34 | Hospital | Ottawa, Canada | TSA (5% SB)/CHR | SAMN26898569 |
| AB218-IK36 | Hospital | Ottawa, Canada | TSA (5% SB)/CHR | SAMN26898570 |
| AB219-IK37 | Hospital | Ottawa, Canada | TSA (5% SB)/CHR | SAMN26898571 |
| AB220-IK38 | Hospital | Ottawa, Canada | TSA (5% SB)/CHR | SAMN26898572 |
| AB223-IK41 | Hospital | Ottawa, Canada | TSA (5% SB)/CHR | SAMN26898573 |
| AB224-IK42 | Hospital | Ottawa, Canada | TSA (5% SB)/CHR | SAMN26898574 |
| AB424-AcS9 | Chlorinated-WWE | Ottawa, Canada | CHR | SAMN26898577 |
| AB425-AcS17 | Post-chlorinated-WWE | Ottawa, Canada | CHR | SAMN26898578 |
| AB426-AcS18 | Post-chlorinated-WWE | Ottawa, Canada | CHR | SAMN26898579 |
| AB427-AcS19 | Post-chlorinated-WWE | Ottawa, Canada | CHR | SAMN26898580 |
| AB428-AcS20 | Post-chlorinated-WWE | Ottawa, Canada | CHR | SAMN26898581 |
| AB429-AcS27 | Chlorinated-WWE | Ottawa, Canada | CHR | SAMN26898582 |
| AB420-MST-SNC-5 | Agricultural surface water | Ottawa, Canada | Karmali media | SAMN26898583 |
| AB421-MST-SNC-9 | Agricultural surface water | Ottawa, Canada | Karmali media | SAMN26898584 |
| AB052-MST-SNC-8 | Agricultural surface water | Ottawa, Canada | Karmali media | SAMN26898585 |
| AB422-MST-SNC-24 | Agricultural surface water | Ottawa, Canada | Karmali media | SAMN26898586 |
| AB423-MST-SNC-253 | Agricultural surface water | Ottawa, Canada | Karmali media | SAMN26898587 |

CHR – CHROMAgar Acinetobacter

Karmali media – *Campylobacter* selective media

SB – Sheep’s Blood

TSA – tryptic soy broth

WWE – waste-water effluent

* As ATCC17978 was received from the ATCC, isolation media is not applicable.

Supplemental Table 3: Novel sequence type and clonal complex assignments.

| Strain | Sequence Type (ST) | Clonal Complex (CC) |
| --- | --- | --- |
| AB426-AcS18 | ST-2633 | CC427 |
| AB428-AcS20 | ST-2634 | Unassigned to known CC |
| AB429-AcS27 | ST-2635 | CC216 |
| AB339-IK13 | ST-2636 | Unassigned to known CC |
| AB340-IK14 | ST-2636 | Unassigned to known CC |
| AB341-IK15 | ST-2636 | Unassigned to known CC |
| AB345-IK29 | ST-2639 | CC132 |
| AB347-IK21 | ST-2640 | Unassigned to known CC |
| AB220-IK38 | ST-2641 | Unassigned to known CC |
| AB224-IK42 | ST-2642 | CC727 |
| AB421-MST-SNC-9 | ST-2643 | Unassigned to known CC |

Supplemental Table 5: Oligonucleotides used in this study for RT-qPCR.

| Target Amplicon | Forward Primer | Reverse Primer |
| --- | --- | --- |
| 16s rRNA | CTTCGGACCTTGCGCTAATA | ATCCTCTCAGACCCGCTACA |
| *adeB* | aatactgccgccaataccag | ggattatggcgactgaagga |
| *adeG* | CGTAACTATGCGGTGCTCAA | ATCGCGTAGTCACCAGAACC |
| *adeJ* | catcggctgaaacagttgaa | gcctgaccattaccagcact |
